# Costs of HIV testing services in sub-Saharan Africa: a systematic literature review

**DOI:** 10.1186/s12879-024-09770-7

**Published:** 2024-08-27

**Authors:** Nurilign Ahmed, Jason J. Ong, Kathleen McGee, Marc d’Elbée, Cheryl Johnson, Valentina Cambiano, Karin Hatzold, Elizabeth L. Corbett, Fern Terris-Prestholt, Hendramoorthy Maheswaran

**Affiliations:** 1 Faculty of Public Health and Policy, London School of Hygiene and Tropical Medicine, 15-17 Tavistock Place, London, WC1H 9SH UK; 2 Faculty of Infectious and Tropical Diseases, London School of Hygiene and Tropical Medicine, London, UK; 3 Central Clinical School, Monash University, Melbourne, Australia; 4 Department of HIV/AIDS, World Health Organization, Geneva, Switzerland; 5 Institute for Global Health, University College London, London, UK; 6 Population Services International, Cape Town, South Africa; 7 Malawi-Liverpool-Wellcome Trust Clinical Research Programme, Blantyre, Malawi; 8 United Nations Joint Programme on HIV AIDS, Geneva, Switzerland; 9 Institute of Global Health Innovation, Imperial College London, London, UK

**Keywords:** Cost, Costing, Cost analysis, HIV testing services, Sub-Saharan Africa

## Abstract

**Objective:**

To review HIV testing services (HTS) costs in sub-Saharan Africa.

**Design:**

A systematic literature review of studies published from January 2006 to October 2020.

**Methods:**

We searched ten electronic databases for studies that reported estimates for cost per person tested ($pptested) and cost per HIV-positive person identified ($ppositive) in sub-Saharan Africa. We explored variations in incremental cost estimates by testing modality (health facility-based, home-based, mobile-service, self-testing, campaign-style, and stand-alone), by primary or secondary/index HTS, and by population (general population, people living with HIV, antenatal care male partner, antenatal care/postnatal women and key populations). All costs are presented in 2019US$.

**Results:**

Sixty-five studies reported 167 cost estimates. Most reported only $pptested (90%), while (10%) reported the $ppositive. Costs were highly skewed. The lowest mean $pptested was self-testing at $12.75 (median = $11.50); primary testing at $16.63 (median = $10.68); in the general population, $14.06 (median = $10.13). The highest costs were in campaign-style at $27.64 (median = $26.70), secondary/index testing at $27.52 (median = $15.85), and antenatal male partner at $47.94 (median = $55.19). Incremental $ppositive was lowest for home-based at $297.09 (median = $246.75); primary testing $352.31 (median = $157.03); in the general population, $262.89 (median: $140.13).

**Conclusion:**

While many studies reported the incremental costs of different HIV testing modalities, few presented full costs. Although the $pptested estimates varied widely, the costs for stand-alone, health facility, home-based, and mobile services were comparable, while substantially higher for campaign-style HTS and the lowest for HIV self-testing. Our review informs policymakers of the affordability of various HTS to ensure universal access to HIV testing.

**Supplementary Information:**

The online version contains supplementary material available at 10.1186/s12879-024-09770-7.

## Research in context

### Evidence before this study

Previous systematic reviews [1–4] have assessed the cost or cost-effectiveness of HIV testing up to 2015. They reported costs for HIV testing modalities across different settings, populations, and contexts. However, there was a gap in systematically assessing the cost of HIV testing services in sub-Saharan African countries to inform policymakers for optimal and affordable HIV testing approaches.

## Added-value of this study

Our study systematically reviewed previous costing studies of HTS in Sub-Saharan Africa. This study adds to previously published SLR by presenting the cost of HTS by country, country income level, country HIV prevalence, cost year, HIV testing modalities, HTS type (direct or secondary index), testing population, and type of cost analysis. We reviewed the cost of HTS to inform HIV testing planning with the most up-to-date economic evidence by including studies published after 2006. We used the Global Health Cost Consortium (GHCC) reference case to assess the quality of the cost studies. This study recommends following the GHCC reference case to standardise the future cost of HIV testing services.

## Implications of all the available evidence

Our findings add to existing publications reviewing the cost studies of HTS in sub-Saharan Africa. This will help policymakers better understand and implement a strategic mix of optimal and affordable HIV testing approaches to accelerate progress toward the 95-95-95 global targets.

## Introduction

HIV continues to be a significant global health concern, affecting 37.7 million people, with 1.5 million newly infected in 2020 [5]. Eastern and Southern Africa continue to be disproportionately affected, accounting for 56% of people living with HIV (PLHIV) globally [5]. The UNAIDS 95-95-95 targets achieve and maintain low HIV incidence by 2030, starting with diagnosing 95% of all PLHIV [6]. While there has been substantial progress, gaps remain with many PLHIV undiagnosed. At the end of 2021, only 90% of PLHIV knew their HIV status in Eastern and Southern Africa [5], with the most significant gaps among key populations, men and adolescents [7–9]. Access to HTS also continues to be an essential part of HIV prevention programs such as voluntary male medical circumcision (VMMC), condoms, harm reduction, and pre-exposure prophylaxis [10–19], which prevent new infections by enabling many people with HIV-related risks to stay negative.

HTSs are widely available in many sub-Saharan African countries, with testing delivered primarily in health facilities (through the outpatient department, antenatal care, Tuberculosis, sexually transmitted infection department) and various other testing modalities such as home-based, workplaces, mobile-service, campaign-style, and stand-alone HTS sites. A range delivers these of healthcare professionals, lay providers and peers, and individuals who may self-test. Together, these strategic approaches can offer a range of options that can reach the PLHIV who do not know their status and those at high ongoing risk who could benefit from prevention, including HIV testing provided through more convenient and confidential approaches like HIV self-testing [2, 20–30]. The sub-Saharan African countries that are striving to reach the first 95 need ways to prioritise limited resources toward the most efficient and effective mix of HTS approaches. There is an urgent need to understand better the costs of different HIV testing modalities to achieve this.

This study systematically reviewed previous costing studies of HTS in sub-Saharan Africa. First, we explored how the costs of different testing modalities varied by the outcome, such as the incremental costs per person tested for HIV and the incremental costs per HIV-positive case identified. Second, we reviewed the incremental cost by different testing modalities, by primary or secondary/index HTS, and by type of population tested.

## Methods

This systematic review followed the Preferred Reporting Items for Systematic Reviews and Meta-Analysis (PRISMA) guidelines (Additional file 1 Table S3) [31]. We limited the review to sub-Saharan Africa. A description of the various HIV testing modalities in sub-Saharan Africa is provided in Table 1 [32]. It categorises the costing studies depending on how the results are presented.

**Table 1:** Definition of the HTS model included in the review [32]

**HTS model**	**Description**
Health facility-based	“HIV testing and counselling (HTC) is a package service intended to allow people to make informed decisions regarding knowledge of their HIV status and the implications of those decisions” [33]. Health facility HIV testing includes the provision of pre-test counselling, HIV rapid tests, and post-test counselling offered to clients within the departments of voluntary counselling and testing (VCT), antenatal clinic (ANC), post-natal care, provider-initiated HIV counselling and testing (PICT) or outpatient department (OPD) or voluntary medical male circumcision (VMMC) centres.
Home-based	Home-based HTS includes pre-test counselling, HIV rapid tests, and post-test counselling by trained HTS providers in the client’s home.
Mobile-service	Mobile HTS uses tents and mobile vans to provide HIV testing in different community locations such as markets, transport hubs, and open fields. The trained HTS provider selects the specific location on an ad hoc basis.
Self-testing	Where a person performs and interprets his or her own HIV test, often in private, self-testing can be done within health facilities or the community or integrated into mobile services or HIV fixed sites or offered at male-dominated workplaces or integrated with VMMC services.
Campaign-style	Ministry of Health or specific organisations uses more accessible community spaces for HIV testing. It is more connected to the community and designed to address community needs.
Stand-alone	Static HTS located near transport hubs and markets where it serves community members.
Other	Primary testing is where HIV testing is provided to the individual accessing the service.
	Secondary testing is where providers offer HIV testing indirectly to an individual’s contacts. This is referred to as Index testing when providers work with individuals living with HIV (index clients) to list and invite their sexual partners for HIV testing and counselling. It is referred to as social network testing when providers approach persons within the same social network for HIV testing and counselling.
	Workplace HIV testing targets industries such as military, mining, agriculture, fishing, and long-distance drivers and offer HIV testing and counselling in the workplace.

### Inclusion and exclusion criteria

Costing studies were eligible for inclusion if they reported any cost estimates for HTS in a sub-Saharan African country. This included cost per person tested (US$pptested) and cost per HIV-positive case identified (US$ppositive). Costing studies were included in the analysis more than once if they reported costs for more than one HIV testing model. We included studies exploring HIV testing in all population groups except those focused on early infant diagnosis. The language was limited to English, including original or translated sources. Additional file 1 Table S1 provides detailed PICOS (Population, Intervention, Comparators, Outcomes, and Study type) detailing the inclusion and exclusion criteria.

### Search strategy and identification of studies

The literature searches were undertaken in December 2019 and updated in October 2020. We searched ten databases: Medline, PubMed, Embase, Popline, Scopus, Global Health, COCHRANE, Social Policy and Practice, Web of Science, and Tuft University cost-effectiveness analysis registry [34]. The search terms were formulated around the following three concepts: (1) HIV, (2) HIV testing (including couples testing and self-testing), and (3) cost and cost-effectiveness analyses. The search strategy included concepts on cost-effectiveness analyses to capture primary costing data used in the cost-effectiveness modelling studies. References of included studies were reviewed for additional relevant articles. For further references, missing outcomes, and clarifications, authors and experts in HIV economics were contacted by e-mail. The full search strategy is included in Additional file 1 Table S2.

### Study selection and data extraction

According to the inclusion criteria, two independent reviewers (NA and KM) independently screened the titles and abstracts for eligibility. Discrepancies were resolved through discussion and consensus by reviewing the full study. N.A. reviewed full studies and created the data extraction template using the Global Health Cost Consortium (GHCC) reference case [35] to characterise eligible studies.

We classified the studies by whether they undertook a cost analysis. Studies were deemed to have conducted a cost analysis if they estimated the costs of delivering the HTS related to the number of HIV tests performed or the number of HIV-positive individuals identified.

### Cost studies

For cost studies, we extracted data on the country of the study, HIV testing modality, costing year, costing perspective, costing method, the total number of HIV tests provided, the total number of HIV-positive cases identified, cost per person tested (US$pptested) and cost per HIV-positive individual identified (US$ppositive). For US$pptested, the total costs of a given HIV testing modality were divided by all individuals that were tested (the sum of the person tested HIV negative and the person tested HIV positive:.

For US$ppositive, the total costs for the given HIV testing modality were divided by all individuals that tested HIV positive (if known, those previously tested positive were excluded): $$\text{US}\$\text{ppositive} = \frac{total\,costs\,for\,HIV \,testing\,services }{\text{Person tested HIV}+}$$. For studies that reported costs for a package of interventions that included HIV testing and other health services (e.g., family planning or tuberculosis screening), we excluded the costs for the other health services delivered. We extracted the year the costing exercise was conducted rather than the year the study was published. We assumed it to be the year before the publication date for studies that did not report the costing year. The included studies reported costing perspectives using different terminologies. We categorised the costing perspective as a provider, patient, or societal. A provider perspective captures the costs incurred by the organisation delivering the health intervention, a patient perspective only includes the costs incurred by the users, and a societal perspective includes all the costs incurred by the organisation, the users and possibly second or third parties affected (e.g. a family member) [36].

We classified the costing methods used at three levels. First, we determined whether the researchers had estimated incremental or full costs. The incremental costs estimate the cost of adding a new health intervention to an existing health program by reporting the additional capital and recurrent costs incurred without accounting for the existing infrastructure and overhead costs borne by the existing health program [37]. An incremental cost analysis may need to be more accurate in determining the cost of delivering new health interventions or the investment needed to sustain the current provision [37]. By contrast, a full cost analysis includes all resources used to introduce the new health intervention, including the infrastructure and overhead costs. Second, we determined whether the costs represent financial or economic costs. Financial costs estimate the actual expenditure on goods and services purchased. Economic costs aim to capture opportunity costs and assess the full value of all resources used, including donated goods and services such as volunteer time, rent, and capital equipment, at market price [38]. Third, we determined whether the cost represented estimates from primary costing studies (referred to as empirical) or modelled costs. Primary costing studies observe actual resource use to estimate costs, whilst modelled costs are based on assumed or expected resource use [38].

### Study quality assessment

Two independent reviewers (NA and MD) assessed the quality of the costing methods using the GHCC reference case [35]. The GHCC comprises 17 principles to guide cost estimation; we assessed whether the study had met these guidelines. A detailed quality assessment for individual studies is included in Additional file 1 Tables S4 & S5.

### Data analysis

All cost estimates were adjusted for inflation using the World Bank’s consumer price index [39] and expressed in 2019 U.S. dollars (US$). First, expenses described in US$ were converted back to the local currency using the World Bank’s exchange rate based on when the cost analysis was done. Second, the cost was inflated using the World Bank’s consumer price index and converted back to US$ using the exchange rate of the base year (2019) [40]. We provide the mean and median estimates for the cost estimates and use the interquartile range (IQR) to reflect the distribution of cost estimates. The boxplot shows the distribution of the cost data based on the five-number summary (minimum cost, first quartile (Q1), median cost, third quartile (Q3), and maximum). The boxplot can inform the outlier costs and values. We did not conduct a meta-analysis on cost estimates due to variations in HTS approaches, populations served, costing perspectives, and methods.

## Results

**Fig. 1:**
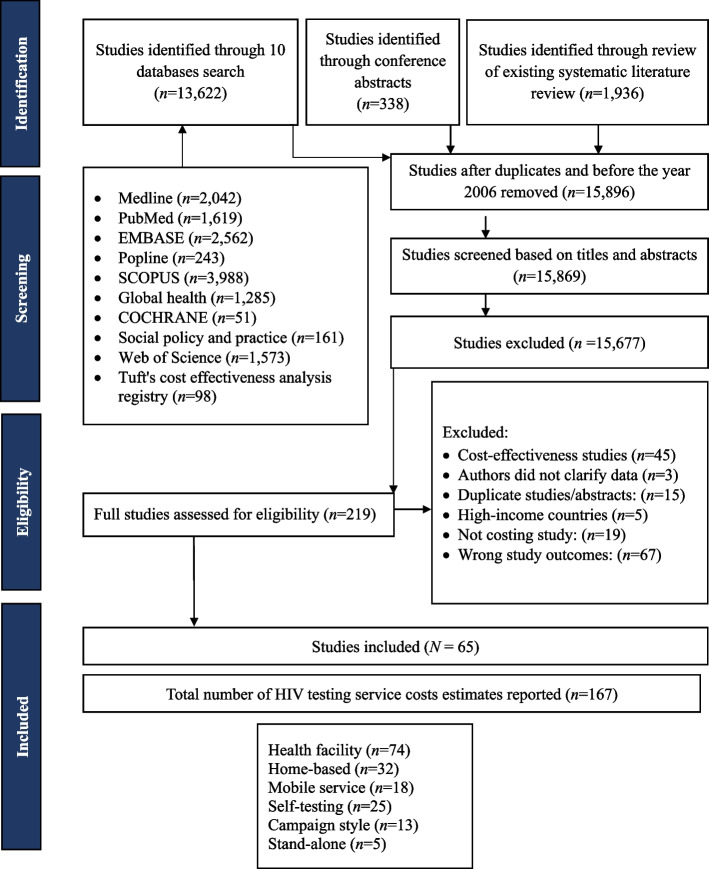
PRISMA flow diagram of the systematic literature review

We identified 65 eligible studies from 26,889 titles and abstracts reviewed. The 65 eligible studies reported 167 cost estimates of HIV testing services. Overall, 74 reported costs for facility-based HTS, 32 for home-based testing, 18 for mobile services, 25 for self-testing, 13 for campaign-style, and 5 for stand-alone HTS (Fig. 1). summarises the results from studies that undertook a cost analysis. Over half of the studies (53%) were conducted in the Southern African region, 41% were conducted in the Eastern African region, and 6% were conducted in West Africa. Studies were undertaken in diverse settings, including low (33%), lower-middle (45%) and upper-middle (22%) -income countries, as well as in low to high HIV prevalent countries (1.2% to 27.1%). Most cost studies reported incremental (77%), financial (47%), and empirical costs (95%). Cost per person tested was reported by 91% of studies; fewer studies reported cost per person tested HIV-positive (56%), and a minority reported cost per person who never tested before (8%) and cost per antiretroviral therapy initiation (14%). No studies on key populations reported the cost per person tested HIV-positive (Table 2). A detailed summary of the cost studies is provided in Additional file 1 Table S6.

**Table 2:** Summary of HTS cost studies undertaken in sub-Saharan Africa between 2006–2020 (*N* = 65)

**Number of studies (N) (%)**	**Cost Estimates (n) (%)**	**Mean incremental cost per person tested ($) (Median IQR) (*n*)**	**Mean incremental cost per positive person tested ($) (Median IQR) (*n*)**
**Total (*N* = 65)**	167 (100%)	$18.45 (median = $12.26, IQR: $7.64–$23.50) (*n* = 124)	$359.76 (median = $168.80, IQR: $80.08–$403.74) (*n* = 71)
**Sub-Saharan African Countries**			
**Kenya (*N* = 11) (17%)**		$25.11 (median = $15.65, IQR: $12.78–$37.33) (*n* = 15)	$178.56(median = $116.80, IQR: $66.84–$168.80) (*n* = 9)
**Malawi (*N* = 9) (14%)**		$13.03 (median = $9.82 IQR: $6.25–$12.84 (*n* = 19)	$149.00 (median = $121.64, IQR: $96.84–$169.16) (*n* = 11)
**South Africa (*N* = 11) (17%)**		$25.91 (median = $13.38, IQR: $7.38–$29.96) (*n* = 22)	$409.78 (median = $156.45, IQR: $19.01–$723.11) (*n* = 23)
**Uganda (*N* = 11) (17%)**		$13.76 (median = $10.95, IQR: $6.43–$15.64) (*n* = 26)	$226.26 (median = $148.40, IQR: $82.10–$246.75) (*n* = 13)
**Zambia (*N* = 5) (8%)**		$21.08 (median = $14.07, IQR: $7.13–$26.43) (*n* = 14)	$345.71 (median = $390.39, IQR: $85.43–$522.94) (*n* = 8)
**Other West African countries (*N* = 4) (6%)**		$22.55 (median = $16.96, IQR: $9.19–$34.09) (*n* = 8)	$1,297.86(median = $931.18, IQR: $444.57–$1,784.47) (*n* = 4)
**Other Southern African countries (*N* = 34) (53%)**		$19.05 (median = $12.13, IQR: $7.29–$19.68) (*n* = 65)	$290.63 (median = $156.45, IQR: $72.26–$403.74) (*n* = 39)
**Other Eastern African countries (*N* = 26) (41%)**		$20.28 (median = $12.44, IQR: $8.11–$18.84) (*n* = 51)	$322.03 (median = $161.60, IQR: $85.38–$339.46) (*n* = 32)
**Country Income Level**			
**Low Income (*N* = 21) (33%)**		$13.97 (median = $10.43, IQR: $6.18–$15.42) (*n* = 50)	$293.71 (median = $144.27, IQR: $92.70–$241.97) (*n* = 26)
**Lower-middle Income (*N* = 29) (45%)**		$19.60 (median = $13.96, IQR: $8.31–$25.76) (*n* = 53)	$393.10 (median = $243.97, IQR: $88.63–$452.96) (*n* = 31)
**Upper-middle Income (*N* = 14) (22%)**		$25.91 (median = $13.38, IQR: $7.38–$29.96) (*n* = 19)	$409.78 (median = $156.45, IQR: $19.01–$723.11) (*n* = 11)
**Country HIV prevalence (year of costing)**			
**< 5% (*N* = 5) (8%)**		$21.05 (median = $13.36, IQR: $9.49–$30.49) (*n* = 13)	$1,374.57 (median = $1,309.58, IQR: $665.45–$1,713.74) (*n* = 6)
**5–10% (*N* = 29) (45%)**		$15.59 (median = $10.95, IQR: $6.39–$16.27) (*n* = 52)	$217.52 (median = $148.40, IQR: $95.79–$237.19) (*n* = 31)
**10–15% (*N* = 15) (23%)**		$17.38 (median = $12.79, IQR: $7.93–$15.83) (*n* = 34)	$225.66 (median = $113.04, IQR: $73.66–$393.67) (*n* = 17)
**15–20% (*N* = 9) (14%)**		$29.99 (median = $23.35, IQR: $7.08–$48.85) (*n* = 17)	$48,125 (median = $356.22, IQR: $22.24–$864.86) (*n* = 11)
**20–25% (*N* = 5) (8%)**		$14.11 (median = $14.08, IQR: $13.72–$14.46) (*n* = 4)	$300.13 (median = $300.13, IQR: $253.42–$346.84) (*n* = 2)
**25–30% (*N* = 1) (2%)**		$11.58 (median = $9.33, IQR: $8.75–$12.16) (*n* = 4)	$205.48 (median = $165.07, IQR: $48.33–$322.21) (*n* = 4)
**Cost Year**			
**2000–2005 (*N* = 3) (5%)**		$15.28 (median = $15.51, IQR: $14.22–$16.57) (*n* = 4)	
**2005–2010 (*N* = 14) (22%)**		$16.34 (median = $11.77, IQR: $8.15–$15.12) (*n* = 27)	$116.91 (median = $94.62, IQR: $48.33–$152.09) (*n* = 16)
**2010–2015(*N* = 25) (39%)**		$20.32 (median = $14.03, IQR: $8.23–$28.63) (*n* = 60)	$465.55 (median = $203.97, IQR: $86.47–$512.75) (*n* = 33)
**2015–2020 (*N* = 22) (34%)**		$17.16 (median = $10.08, IQR: $5.73–$14.23) (*n* = 34)	$377.69 (median = $352.88, IQR: $131.77–$506.29) (*n* = 19)
**HTS Modality**			
**Campaign style (*N* = 5) (8%)**		$27.64 (median = $26.70, IQR: $12.42–$41.93) (*n* = 4)	$413.14 (median = $388.70, IQR: $258.16–$555.91) (*n* = 3)
**Health facility based (*N* = 34) (53%)**		$19.63 (median = $10.70, IQR: $6.00–$28.63) (*n* = 56)	$398.95 (median = $148.29, IQR: $69.85–$429.42) (*n* = 32)
***ANC/PMTCT* (*N* = 6) (18%)**		$42.74 (median = $46.75, IQR: $16.24–$66.62) (*n* = 9)	$967.23 (median = $518.84, IQR: $399.42–$1,039.32) (*n* = 8)
***VCT* (*N* = 16) (47%)**		$14.68 (median = $10.71, IQR: $6.18–$16.02) (*n* = 26)	$276.35 (median = $122.62, IQR: $72.96–$171.74) (*n* = 16)
***Integrated* (*N* = 5) (15%)**		$33.77 (median = $18.40, IQR: $14.21–$47.91) (*n* = 7)	$19.31 (median = $19.31, IQR: $19.31–$19.31) (*n* = 3)
***OPD* (*N* = 7) (21%)**		$6.91 (median = $6.53, IQR: $3.14–$8.09) (*n* = 13)	$83.96 (median = $66.84, IQR: $35.45–$125.31) (*n* = 5)
**Home-based *(N* = 13) (20%)**		$19.30 (median = $13.42, IQR: $8.34–$23.35) (*n* = 29)	$297.09 (median = $246.75, IQR: $132.60–$381.62) (*n* = 15)
**Mobile service (*N* = 5) (8%)**		$16.47 (median = $12.88, IQR: $9.88–$23.94) (*n* = 13)	$356.93 (median = $206.71, IQR: $126.32–$387.29) (*n* = 11)
**Self-testing**^**d**^** (*N* = 6) (9%)**		$12.75 (median = $11.50, IQR: $9.27–$13.92) (*n* = 19)	$338.57 (median = $113.04, IQR: $78.06–$516.30) (*n* = 9)
***Community based* (*N* = 1) (17%)**		$9.83 (median = $9.84, IQR: $5.48–$14.17) (*n* = 6)	$529.59 (median = $529.59, IQR: $522.94–$536.23) (*n* = 2)
***Facility based* (*N* = 4) (67%)**		$10.70 (median = $10.55, IQR: $10.18–$12.25) (*n* = 9)	$92.00 (median = $83.32, IQR: $44.12–$106.92) (*n* = 6)
***Home-based* (*N* = 1) (17%)**		$21.76 (median = $14.03, IQR: $12.83–$22.96) (*n* = 4)	$1,435.94 (median = $1,435.94, IQR: $1,435.94–$1,435.94) (*n* = 1)
**Stand-alone (*N* = 1) (2%)**		$20.61 (median = $20.52, IQR: $15.10–$26.08) (*n* = 3)	$107.15 (median = $107.15, IQR: $107.15–$107.15) (*n* = 1)
**HTS type**			
***Direct* (*N* = 57) (89%)**		$16.71 (median = $10.95, IQR: $7.24–$18.72) (*n* = 104)	$340.16 (median = $161.60, IQR: $79.07–$393.64) (*n* = 66)
***Secondary/Index***^***a***^** (*N* = 7) (11%)**		$27.52 (median = $15.85, IQR: $14.41–$38.88) (*n* = 20)	$618.48 (median = $356.22, IQR: $246.75–$1,041.58) (*n* = 5)
**Testing population**			
**General population(s)**^**b**^** (*N* = 10) (16%)**		$14.39 (median = $10.25, IQR: $7.00–$15.52) (*n* = 92)	$255.40(median = $148.40, IQR: $72.26–$348.18) (*n* = 59)
**PLHIV Partners (*N* = 3) (5%)**		$19.31 (median = $15.57, IQR: $14.86–$27.09) (*n* = 14)	$246.75 (median = $246.75, IQR: $246.75–$246.75) (*n* = 1)
**ANC/PMTCT Male Partners (*N* = 3) (5%)**		$47.94 (median = $49.17, IQR: $13.39–$55.19) (*n* = 5)	$711.41 (median = $698.90, IQR: $270.14–$1,140.17) (*n* = 4)
**Pregnant women or women breastfeeding (*N* = 5) (8%)**		$39.25 (median = $41.32, IQR: $14.08–$62.39) (*n* = 10)	$1,054.52 (median = $524, IQR: $463.28–$1,300.53) (*n* = 7)
**Key Population(s)**^**c**^** (*N* = 2) (3%)**		$20.31 (median = $9.49, IQR: $8.00–$27.21) (*n* = 3)	–
**Type of Cost Analysis**			
**Incremental vs. Full (*N* = 49 vs. 15) (77% vs.23%)**	Incremental	$18.45 (median = $12.26, IQR: $7.64–$23.50) (*n* = 124)	$359.76 (median = $168.80, IQR: $80.08–$403.73) (*n* = 71)
	Full	$38.65 (median = $32.83, IQR: $25.47–$45.69) (*n* = 33)	$367.43 (median = $322.92, IQR: $85.22–$582.91) (*n* = 16)
**Financial vs. Economic (*N* = 30 vs. 34) (47% vs.53%)**	Financial	$19.13 (median = $13.11, IQR: $7.52–$12.88) (*n* = 72)	$334.37 (median = $237.19, IQR: $79.28–$449.47) (*n* = 40)
	Economic	$25.71 (median = $15.97, IQR: $9.82–$35.00) (*n* = 85)	$383.98 (median = $157.03, IQR: $82.27–$494.50) (*n* = 47)
**Empirical vs. Modelled (*N* = 61 vs. 3) (95% vs.5%)**	Empirical	$22.96 (median = $14.49, IQR: $8.76–$31.53) (*n* = 154)	$363.93 (median = $177.58, IQR: $81.09–$474.91) (*n* = 84)
	Modelled	$9.01(median = $7.60, IQR: $5.49–$11.82) (*n* = 3)	$283.75 (median = $349.54, IQR: $187.50–$412.89) (*n* = 3)

Western A.U. countries (Nigeria) (*N* = 4), Southern AU countries (Botswana, Eswatini, Lesotho, Namibia, South Africa, Zambia, Zimbabwe *(N* = 35), Eastern A.U. countries (Ethiopia, Kenya, Rwanda, Tanzania, Uganda (*N* = 26)

^a^Secondary index testing focused on testing sexual partner(s) of HIV-positive individuals

^b^General population represented those people considered at risk of HIV acquisition and therefore deserving of HIV testing

^c^No study reported cost per positive case identified for key populations. “UNAIDS considers gay men and other men who have sex with men, sex workers, transgender people, people who inject drugs and prisoners and other incarcerated people as the five main key population groups that are particularly vulnerable to HIV and frequently lack adequate access to services.” Male truckers would fall into UNAID’s definition of vulnerable populations but not key populations[41]

^d^The cost per positive case identified includes the cost of confirmatory testing for those who reported positive HIV self-testing, except for one study [42], which was not clearly stated

### Cost analysis

**Fig. 2:**
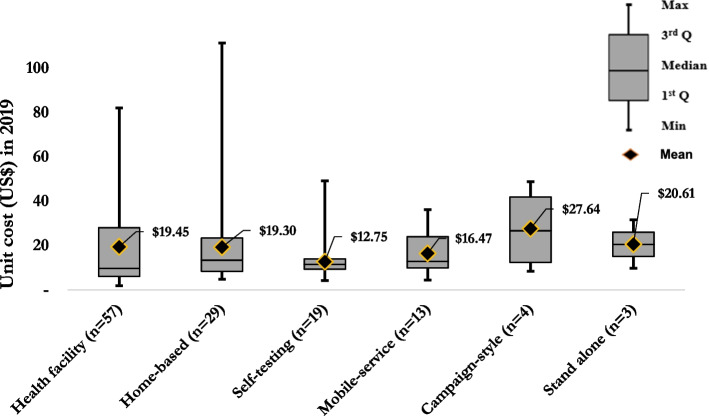
Mean and distribution of the incremental cost per person tested by mode of HTS in 2019 US$

Figure 2 shows the incremental cost estimates for US$pptested by HIV testing modalities from the provider’s perspective. The mean cost estimate for self-testing was $12.75 (median = $11.50, IQR: $9.27–$13.92) [43–45]; for mobile-services was $16.47 (median = $12.88, IQR: $9.88–$23.94) [46–55]; for home-based testing was $19.30 (median = $13.42, IQR: $8.34–$23.36) [50, 53–66]; facility-based HTS was $19.63 (median = $10.70, IQR: $6.00–$28.63) [10, 46, 54, 60–62, 65, 67–81]; for stand-alone HTS was $20.61 (median = $20.52, IQR: $15.10–26.08) [49, 60], and for campaign-style was $27.64 (median = $26.70, IQR: $12.42–$41.93) [52, 82, 83]. Most cost estimates were for facility-based testing (*n* = 74), with only 13 estimates for campaign-style HTS (Fig. 2).

**Fig. 3:**
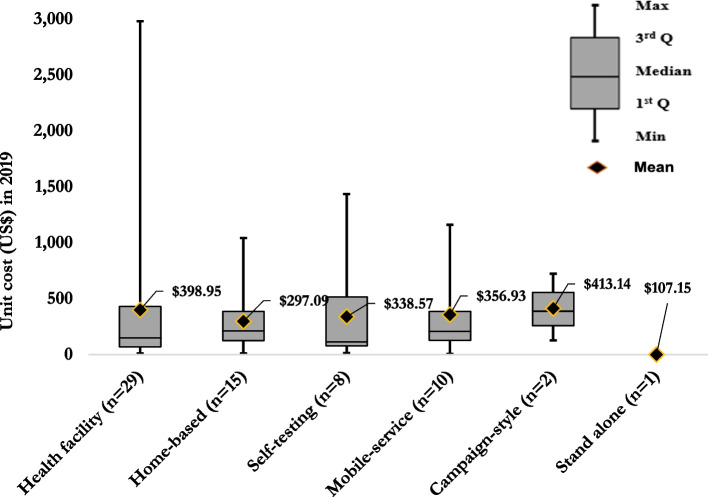
Mean and distribution of the incremental cost per person tested positive by mode of HTS in 2019 US$

Figure 3 shows the incremental estimates for US$ppositive by testing modality. The mean cost estimate for home-based testing was $297.09 (median = $246.75, IQR: $132.60–$381.62) [50, 53, 55, 57–60, 62–64, 66]; for self-testing, it was $338.57 (median = $113.04, IQR: $78.06–$516.30) [44]; for mobile-services was $356.93 (median = $206.71, IQR:$126.321–$387.29) [48–53, 55, 59, 68]; for facility-based HTS was US$398.95 (median = $148.29, IQR: $69.85–$429.42) [60, 62, 68, 69, 71, 73, 79, 81]; and for campaign-style was $413.14 (median = $388.70, IQR: $258.16–$555.91) [52]. Only one study estimated the US$ppositive for stand-alone HTS and found it to be $107.15 [60] (Fig. 3).

**Fig. 4:**
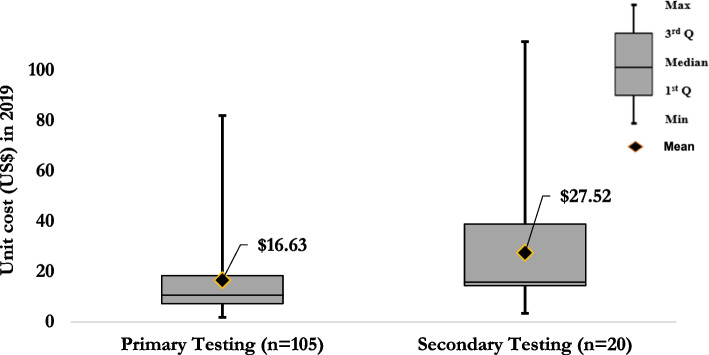
Mean and distribution of the incremental cost per person tested by primary/direct or secondary/index HTS in 2019 US$

For the direct/primary HIV testing services, the mean estimate for the incremental US$pptested was $16.63 (median = $10.68, IQR: $7.29–$18.40) [12, 43–46, 48–55, 57–66, 68–73, 75, 76, 79, 81, 84–105], whilst for secondary/index HIV testing, the mean estimate for the incremental US$pptested was $27.52 (median = $15.85, IQR: $14.41–$38.88) [42, 60, 67, 78, 80, 106–108] (Fig. 4).

**Fig. 5:**
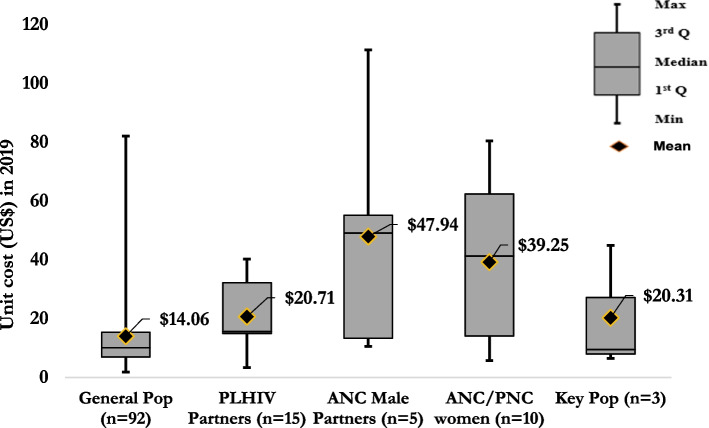
Mean and distribution of the incremental cost per person tested by population tested in 2019 US$

Figure 5 shows the incremental US$pptested by type of population tested. For the general population, the mean estimate for the incremental US$pptested was $14.06 (median = $10.13, IQR: $7.00–$15.42); for PLHIV partners, $19.31 (median = $15.57, IQR: $14.86–$27.09); for key populations $20.31 (median = $9.49, IQR: $8.00–$27.21), for ANC/ Post-Natal Care $39.28 (median = $41.32, IQR: $14.08–$62.39); and for ANC partners $47.94 (median = $49.17, IQR: $13.39–$55.19) (Fig. 5).

**Fig. 6:**
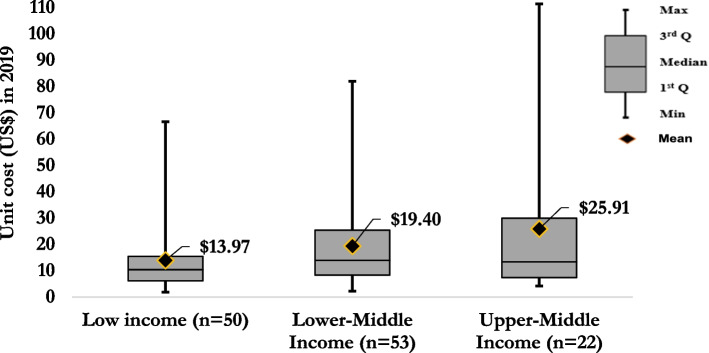
Mean and distribution of the incremental cost per person tested by country income level in 2019 US$

Figure 6 shows the incremental US$pptested by country income level. For low-income, the mean estimate for the incremental US$pptested was $13.97 (median = $10.43, IQR: $6.18–15.42), for lower-middle-income $19.40 (median = $13.96, IQR: $8.31–25.76) and upper-middle-income $25.91 (median = 13.38, IQR: $7.38–29.96) (Fig. 6).

**Fig. 7:**
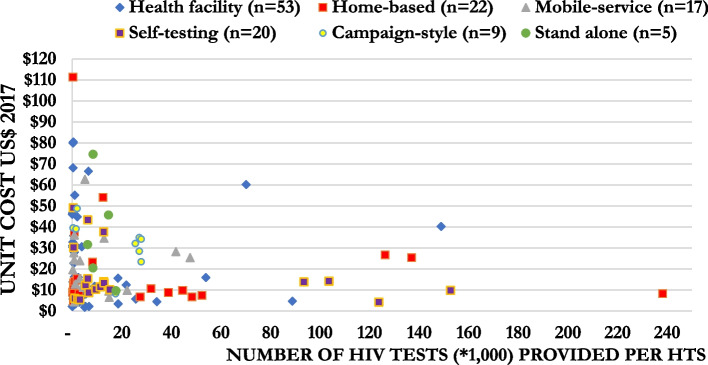
Mean and distribution of the incremental cost per person tested by the number of persons tested by mode of HTS in 2019 US$

Figure 7 shows the incremental US$pptested by the scale of the HTS cost, represented by the number of tests performed during their analysis. For HTS where less than 10,000 HIV tests were provided, the mean estimate for the incremental US$pptested was $23.06 (median = $14.45, IQR: $7.89–31.31), for those that provided between 10,000 and 20,000 HIV tests it was $25.67 (median = $22.01, IQR: $3.43–34.78), and for those that provided greater than 20,000 HIV tests it was $18.22 (median = $13.84, IQR: $4.25–26.77) (Fig. 7).

**Fig. 8:**
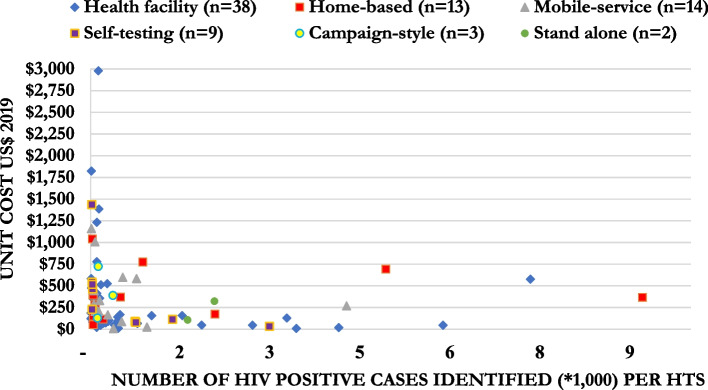
Mean and distribution of the incremental cost per person tested by the number of persons tested positive by mode of HTS in 2019 US$

Figure 8 shows the mean incremental US$ppositive by the scale of the HTS, represented by the number of HIV-positive individuals identified. For HTS services where less than 1,000 HIV-positive individuals were identified, the mean estimate for the incremental US$ppositive was $428.08 (median = $263.99, IQR: $95.08–522.78). For HTS that identified between 1,000 and 5,000 HIV-positive individuals, the mean estimate for the incremental US$ppositive was $154.58 (median = $113.04, IQR: $9.69–157.03), and for HTS that identified greater than 5,000 HIV-positive individuals, it was $329.93 (median = $366.97, IQR: $206.44–471.94). These figures suggest economies of scale where costs are lower in larger-scale testing programmes and reactivity rates are higher (Fig. 8).

The mean estimate for the incremental costs were $18.45 (median = $12.26, IQR: $7.64–$23.50) for cost per person tested and $359.76(median = $168.80, IQR: $80.08–$403.74) for cost per HIV-positive individual identified. The mean estimate for the full costs (where costs incurred to introduce the new intervention are included) were $38.65 (median = $32.83, IQR: $25.47–$45.69) for cost per person tested and $367.43 (median = $322.92, IQR: $85.22–$582.91) for cost per HIV-positive person identified (Additional file 1 Table S5).

Tables 3 and 4 show the quality assessment of the cost studies and their compliance with the 17 principles of the GHCC reference case [109]. Most cost studies complied with principles 1 to 13 and 17 and did not fully comply with principles 14 to 16 of the GHCC reference case (Additional file 1 Tables S4). The three relate to whether authors sufficiently accounted for the opportunity cost of volunteer time (Principal 14), explored variation in costs (Principal 15), or undertook sensitivity analysis to characterise uncertainty in their estimates (Principal 16).

**Table 3:** Quality assessment: Percentage of the cost studies compliant with GHCC Reference Case^a^

Quality assessment of cost studies (*N* = 65) following the GHCC principles in %									
**Reported cost estimated by testing modality**	**Study purpose and population (P1)**	**Study perspective and types of costing approach used (P2-3)**	**Unit cost, time horizon, scope, the quantity of inputs, sampling, and data source strategy (P4-9)**	**Timing of data collection sources for price data (P10-11)**	**Annualisation or depreciation of capital cost and discounting (P12-13)**	**Shadow prices for goods and for the opportunity cost of time (P14)**	**Characterising heterogeneity (P15)**	**Characterising uncertainty (P16)**	**Communicated limitations, conflicts of interest (P17)**
Health facility (*n* = 76)	100%	80%	73%	87%	87%	22%	26%	17%	91%
Home-based (*n* = 32)	100%	85%	77%	88%	77%	8%	8%	31%	100%
Mobile services (*n* = 18)	100%	93%	91%	100%	86%	0%	14%	71%	100%
Self-testing (*n* = 25)	100%	100%	100%	100%	100%	33%	33%	100%	100%
Campaign style (*n* = 13)	100%	100%	100%	100%	100%	0%	50%	50%	100%
Stand-alone (*n* = 5)	100%	100%	83%	50%	100%	0%	0%	0%	100%

^a^Data are presented as % unless otherwise indicated

**Table 4:** Findings from a quality assessment using the GHCC’s principles and methods reporting checklist for cost studies included in the review [109] (*N* = 65)

**Author, year (Ref)**	**P1**	**P2**	**P3**	**P4**	**P5**	**P6**	**P7**	**P8**	**P9**	**P10**	**P11**	**P12**	**P13**^**a**^	**P14**	**P15**	**P16**	**P17**	**Source**	**Score**^**a**^
Adebajo, 2013 [46]	Y	N	N	N	N	N	N	N	N	N	N	N/A	N/A	N	N	N	N	Slides	3/17
Ahmed, 2018 [43]	Y	Y	Y	Y	Y	Y	Y	Y	Y	Y	Y	N/A	N/A	N	N	Y	Y	Poster	15/17
Aliyu, 2012 [84]	Y	Y	Y	Y	Y	Y	Y	Y	Y	Y	Y	N/A	N/A	N	Y	N	Y	PRP	15/17
Allen, 2014 [67]	Y	Y	N	N	Y	N	N	N	N	N	Y	N	N/A	N	N	N	N	Abstract	5/17
Bassett, 2007 [68]	Y	Y	Y	Y	Y	Y	Y	Y	Y	Y	N	N/A	N	N	N	N	Y	PRP	12/17
Bassett, 2014 [47]	Y	Y	N	Y	Y	Y	Y	Y	N	Y	Y	N/A	Y	N	N	Y	Y	PRP	13/17
Bautista-Arredondo, 2016 [69]	Y	Y	Y	Y	Y	Y	Y	Y	Y	Y	Y	N/A	N/A	Y	Y	N	Y	PRP	16/17
Bautista-Arredondo, 2018 [110]	Y	Y	N	Y	Y	Y	Y	Y	Y	Y	Y	N	N	Y	Y	N	Y	PRP	13/17
Bogart, 2017 [85]	Y	Y	Y	Y	Y	Y	Y	Y	Y	Y	Y	N/A	N/A	Y	Y	N	Y	PRP	16/17
Bulterys, 2020 [106]	Y	Y	Y	Y	Y	Y	Y	Y	Y	Y	Y	Y	Y	N	N	Y	Y	PRP	15/17
Cham, 2019 [86]	Y	Y	Y	Y	Y	N	N	Y	Y	Y	Y	Y	Y	N	Y	N	Y		13/17
Change, 2016 [48]	Y	Y	Y	Y	Y	Y	Y	Y	Y	Y	Y	N/A	N/A	N	N	N	Y	PRP	14/17
Cherutich, 2018 [107]	Y	Y	Y	Y	Y	Y	Y	Y	Y	N	Y	N/A	Y	N	Y	Y	Y	PRP	14/17
deBeer, 2015 [111]	Y	Y	Y	Y	Y	Y	Y	Y	Y	Y	Y	Y	Y	Y	Y	Y	Y	PRP	17/17
d’Elbée, 2020 [87]	Y	Y	Y	Y	Y	Y	Y	Y	Y	Y	Y	Y	Y	Y	Y	Y	Y	PRP	17/17
George, 2018 [88]	Y	Y	Y	Y	Y	Y	Y	Y	Y	Y	Y	Y	Y	Y	Y	Y	Y	PRP	17/17
Golovaty, 2018 [89]	Y	Y	Y	Y	Y	Y	Y	Y	N	Y	N	Y	Y	N	N	Y	Y	PRP	13/17
Grabbe, 2010 [49]	Y	Y	Y	Y	Y	Y	Y	Y	Y	Y	Y	Y	Y	N	N	N	Y	PRP	14/17
Hauck, 2018 [57]	Y	Y	Y	Y	Y	Y	Y	Y	Y	Y	Y	N/A	N/A	N	N	Y	Y	Slides	15/17
Hausler, 2006 [70]	Y	Y	Y	Y	Y	Y	Y	Y	Y	Y	Y	Y	Y	N	N	N	Y	PRP	14/17
Helleringer, 2013 [58]	Y	Y	Y	Y	Y	Y	Y	Y	Y	Y	Y	N/A	N/A	N	N	N	Y	PRP	14/17
Hewett, 2016 [90]	Y	N	N	Y	N	N	N	Y	N	N	N	N	N	N	Y	N	Y	PRP	5/17
Ibekwe, 2017 [71]	Y	N	N	Y	N	N	N	N	N	N	N	N/A	N/A	N	N	N	N	Abstract	4/17
Kabami, 2017 [48]	Y	N	Y	Y	N	Y	N	Y	Y	N	Y	Y	Y	Y	Y	N	Y	PRP	12/17
Kahn, 2011 [82]	Y	Y	Y	Y	Y	Y	Y	Y	Y	Y	Y	Y	Y	N	N	N	Y	PRP	14/17
Kahwa, 2008 [93]	Y	Y	Y	Y	Y	Y	Y	Y	Y	Y	Y	Y	N/A	N	N	N	Y	PRP	14/17
Korte, 2020 [42]	Y	Y	Y	Y	Y	N	N	Y	Y	Y	N	N	N	N	N	N	Y	PRP	9/17
Labhardt, 2014 [50]	Y	Y	Y	Y	Y	Y	Y	Y	Y	Y	Y	Y	Y	N	Y	Y	Y	PRP	16/17
Labhardt, 2019 [94]	Y	N	N	Y	Y	N	N	Y	Y	Y	N	N	N	N	Y	N	Y	PRP	8/17
Lasry, 2019 [59]	Y	Y	Y	Y	Y	Y	Y	Y	Y	Y	Y	N/A	N/A	N	N	Y	Y	PRP	15/17
Liambila, 2008 [72]	Y	Y	Y	Y	Y	Y	Y	Y	Y	Y	Y	N/A	N/A	N	Y	N	Y	Report	15/17
Maheswaran, 2016 [44]	Y	Y	Y	Y	Y	Y	Y	Y	Y	Y	Y	Y	N/A	Y	Y	Y	Y	PRP	17/17
Maheswaran, 2017 [112]	Y	Y	Y	Y	Y	Y	Y	Y	Y	Y	Y	Y	N/A	Y	Y	Y	Y	PRP	17/17
Meehan, 2017 [52]	Y	Y	Y	Y	N	Y	Y	Y	Y	Y	Y	Y	Y	N	N	N	Y	PRP	13/17
Mangenah, 2019 [45]	Y	Y	Y	Y	Y	Y	Y	Y	Y	Y	Y	N/A	N/A	N	N	Y	Y	PRP	15/17
Medley, 2019 [59]	Y	N	N	N	Y	N	N	N	N	Y	N	N	N	N	Y	N	N	Abstract	4/17
Menzies, 2009 [60]	Y	Y	Y	Y	Y	Y	Y	Y	N	Y	N	N/A	N/A	N	N	N	Y	PRP	12/17
Mostert, 2020 [113]	Y	Y	Y	Y	Y	N	N	Y	N	Y	N	N	N	N	Y	N	N	Abstract	8/17
Muhumuza, 2012 [61]	Y	N	N	Y	Y	N	N	N	N	Y	N	N	N	N	N	N	Y	Abstract	5/17
Mulogo, 2013 [62]	Y	Y	Y	Y	Y	Y	Y	Y	Y	Y	Y	N/A	N/A	N	N	N	Y	PRP	14/17
Mwenge, 2017 [73]	Y	Y	Y	Y	Y	Y	Y	Y	Y	Y	Y	N/A	N/A	N	N	Y	Y	PRP	15/17
Negin, 2009 [63]	Y	N	Y	Y	Y	Y	N	Y	N	Y	Y	N/A	N/A	N	N	N	Y	PRP	11/17
Nichols, 2020 [99]	Y	Y	N	Y	Y	N	Y	Y	Y	N	Y	N	Y	N	N/A	Y	Y	PRP	12/17
Nichols, 2019 [114]	Y	Y	Y	N	Y	N	N	Y	N	Y	N	N	N	N	Y	N	N	Abstract	7/17
Obure, 2012 [75]	Y	Y	Y	Y	Y	Y	Y	Y	Y	Y	Y	Y	N/A	Y	N	N	Y	PRP	16/17
Obure, 2015 [74]	Y	Y	Y	Y	Y	Y	Y	Y	Y	Y	Y	Y	N/A	Y	N	N	Y	PRP	15/17
Ochoa-Moreno, 2020 [100]	Y	Y	Y	Y	Y	Y	Y	Y	Y	Y	Y	N/A	N	Y	Y	Y	Y	PRP	16/17
Orlando, 2010 [13]	Y	Y	Y	Y	Y	Y	Y	Y	Y	Y	Y	Y	N/A	N	N	Y	Y	PRP	15/17
Parker, 2015 [53]	Y	Y	Y	Y	Y	Y	Y	N	N	Y	Y	N	N	N	N	N	Y	PRP	10/17
Perchal, 2006 [76]	Y	N	Y	Y	Y	N	Y	N	Y	Y	Y	N/A	N/A	N	N	N	Y	Slides	11/17
Perez, 2016 [54]	Y	Y	Y	Y	Y	Y	Y	Y	Y	Y	Y	N/A	N/A	N	N	N	Y	Poster	14/17
Rutstein, 2013 [78]	Y	Y	Y	Y	Y	Y	Y	Y	Y	Y	Y	N/A	N/A	N	N	N	Y	PRP	14/17
Settumba, 2015 [102]	Y	Y	Y	Y	Y	Y	N	Y	Y	Y	Y	Y	Y	N	Y	N	Y	PRP	14/17
Shade, 2013 [79]	Y	Y	Y	Y	Y	Y	Y	Y	Y	Y	Y	N/A	N/A	N	Y	N	Y	PRP	15/17
Sharma, 2016 [80]	Y	Y	Y	Y	Y	Y	Y	Y	Y	Y	Y	Y	Y	N	Y	Y	Y	PRP	16/17
Sharma, 2014 [55]	Y	Y	N	Y	N	Y	N	Y	Y	Y	N	N	N	N	N	N	Y	Abstract	8/17
Smith, 2015 [64]	Y	Y	Y	Y	Y	Y	Y	Y	Y	Y	Y	N/A	N/A	N	N	Y	Y	PRP	15/17
Tabana, 2015 [65]	Y	Y	Y	Y	Y	Y	Y	Y	Y	Y	Y	Y	Y	N	N	Y	Y	PRP	15/17
Terris-Prestholt, 2006 [103]	Y	Y	Y	Y	Y	Y	Y	Y	Y	Y	Y	Y	Y	N	Y	Y	Y	PRP	16/17
Terris-Prestholt, 2008 [81]	Y	Y	Y	Y	Y	Y	Y	Y	Y	Y	Y	Y	Y	Y	Y	Y	Y	PRP	14/17
Toure, 2013 [104]	Y	Y	Y	Y	N	Y	Y	Y	Y	Y	Y	Y	Y	Y	N/A	N	Y		15/17
Tumwesigye,2010 [66]	Y	Y	Y	Y	Y	N	N	Y	N	Y	N	Y	Y	Y	N	N	Y	PRP	11/17
Vyas, 2021 [115]	Y	Y	Y	Y	Y	Y	Y	N/A	Y	Y	Y	Y	Y	Y	N/A	Y	Y	PRP	17/17
Vyas, 2020 [105]	Y	Y	Y	Y	Y	Y	Y	N/A	Y	Y	Y	Y	Y	Y	N/A	Y	Y	PRP	17/17

*PRP* Peer-reviewed papers

^a^Non applicable = N/A was assigned to discount if the analysis was limited to one year. Additional points were awarded to the “Score” column if the study’s cost principle(s) was/were N/A

## Discussion

This review adds to existing systematic literature reviews of HIV testing [1–4] by synthesising the costs of HIV testing strategies in sub-Saharan Africa from 2006 until the end of 2020. This study aims to show policymakers the difference in cost for different HIV testing strategies so that policymakers can implement a strategic mix of optimal and affordable HIV testing approaches to accelerate progress toward the 95-95-95 global targets.

We identified cost estimates for six different HIV testing modalities. We found the incremental costs to test individuals through stand-alone, health facility, home-based, and mobile services were comparable (Fig. 2). In contrast, the incremental costs were substantially higher for campaign-style at a mean of $27.64 (median = $26.70, IQR $12.42–$41.93) and lower for HIV self-testing at $12.75 (median = $11.50, IQR $9.27–$13.92) per person tested. The mean incremental costs for facility-based testing $19.63 (median = $10.70, IQR $6.00–$28.63) and home-based testing $19.30 (median = $13.42, IQR $8.34–$23.35) were similar. This could be explained by the fact that the number of people tested for home-based testing is much higher (36,377) than facility-based testing (10,722), which may have reduced the mean incremental costs. This could also explain the difference in resource use or a methodological difference in how the studies presented their costs. Despite differentiating between full and incremental costs, cost variances across studies are significant, particularly for facility-based HTS (range: $1.82–$82.04), home-based (range $4.75–$111.38), and self-testing (range: $4.25–$49.17) due to the heterogeneity of the scope of the costing studies.

The incremental costs per person tested through secondary/index HIV testing services, $27.52 (median = $15.85, IQR:$14.41–$38.88), were higher than the incremental costs per person tested through primary/direct HIV testing services, $16.71 (median = $10.68, IQR:$7.29–$18.40). The mean number of persons tested in the direct HTS was 20,445 compared with 13,638 in the secondary/index HTS across all studies and testing modalities. This study also found that the incremental cost per person testing through ANC testing, $42.74 (median = $46.75, IQR: $16.24–$66.62), is much more expensive than other HTS modalities where we found the mean number of persons tested in ANC was the lowest at 4,418 compared with other HTS modalities. This is a potential reason for the discrepancy in cost per person tested. ANC and secondary/index testing can potentially improve testing uptake amongst children and men [116, 117], and their costs should be further explored.

The cost per HIV-positive individual identified were varied across the six HIV testing modalities. Across the studies, the mean estimate for the incremental cost per HIV-positive identified at the health facility, home-based, self-testing, and mobile services were $398.95, $297.09, $338.57 and $356.57, respectively. Although there were a small number of cost estimates for campaign-style (*n* = 13) and stand-alone (*n* = 2) HIV testing modalities, the mean costs were $413.14 and $107.15 per HIV-positive identified, respectively. Interpreting these cost estimates should be done with caution. Variations in HIV prevalence likely explained some differences observed in cost estimates, the number of people tested, and the number of positive cases identified across settings. For example, low HIV prevalence and high HIV testing rates in Rwanda led to low yields and higher costs per HIV-positive person identified [69]. This may contribute to greater overall through earlier treatment and care initiation to improve individual and population level benefits. One study presented cost estimates for two rounds of home-based HIV testing and reported the cost per HIV-positive person identified nearly doubled between the two rounds (first round $366.97 vs second round $691.82), and a reduction in HIV positivity rate partly explained this [57]. The authors also stated costs were sensitive to community-specific factors such as service delivery and population characteristics [57]. Thus, strategies including HIVST and door-to-door testing every 3–5 years may be a way to maximise limited resources. This review identified no studies that reported cost per positive case identified for the key populations. However, several key population programmes are focused on prevention strategies. Thus, it is important to calculate the cost per case identified for the key populations to inform better transitions for antiretroviral therapy and other prevention strategies.

When looking at the cost studies by type of population tested, the mean incremental cost per person tested was lowest amongst the general population at $14.39 (median = $10.25, IQR: $7.00–$15.52) and the highest for testing more targeted populations, especially for ANC male partners $47.94 (median = $49.17, IQR: $13.39–$55.19) and women in antenatal or postnatal care $39.25 (median = $41.32, IQR: $14.08–$62.39). ANC male partners and secondary/index testing are more targeted approaches that yield greater testing volume. The provision of testing for ANC male partners and secondary/index testing is not just about the cost per case identified; it yields prevention benefits and contributes to eliminates mother-to-child HIV transmission. Based on the studies reviewed, these were also the most affordable, considering greater yield. One of the reasons the costs between specific populations and general populations cannot easily be compared is the heterogeneity of the HIV epidemics, where HIV prevalence and HIV testing are different between the specific populations and the general populations. One of the limitations of secondary/index testing is that the cost per case identified is higher when HIV testing of male partners includes post-test counselling on the phone and incentives (e.g., airtime vouchers) (Medley 2019). The effect of this would be higher costs without parsing out the impact of strategies that included a much larger sample of children and those that were adults (key or general population). However, it was not feasible to address these in our analysis due to data scarcity and exclusion criteria. This review identified that HIVST might be a promising way to reduce costs while other HTS are high, as it is one of the lowest-cost options.

When looking at the cost studies by country income level, the mean incremental cost per person tested increased along with countries’ income ranging from $13.97 (median = $10.43, IQR: $6.18–$15.42) for low income to $19.60 (median = $13.96, IQR: $8.31–$25.76) for lower-middle-income and $25.91 (median = $13.38, IQR: $7.38–$29.96) for upper-middle-income. These costs should not be generalised; for example, heterogeneity of the studies could vary the cost.

For policy makers, the choice of one testing modality over another could be driven by which HIV testing approach is most feasible to implement and most likely to reach their untested and under-served populations. Additionally, this study’s cost findings may encourage policymakers to consider delivering a mixture of testing modalities. However, this needs to be considered in the context of losing potential economies of scale from delivering larger single model HTS. Policymakers and implementing partners would find the result of economies of scale as evidence to scale up a larger single model HTS to lower costs. Figures 7 and 8 showed potential economies of scale where the provision of more HIV testing could help spread overhead costs and lead to reducing cost per person tested and cost per case identified. Figures 7 and 8 also showed the economies of scale of all six HIV testing modalities. However, this finding should be interpreted with caution given the heterogeneity of the studies. If the HTS aims to reach a population of first-time testers to increase HIV diagnosis and antiretroviral therapy initiation, scaling up the HTS is encouraged to lower the costs. However, it is critical to recognise that to reach the last few percentiles of first-time testers, the provision of HTS to identify the last few HIV-positive cases would likely result in diseconomies of scale, and costs will rise. Moreover, adding choice to the testing campaigns, shown by d’Elbée et al. in Lesotho [87], it can increase the number of people linked to antiretroviral therapy.

We observed variations in costing methods that reported incremental vs full cost or financial vs economic cost estimates. Most studies estimated the incremental costs. We found that the estimated incremental costs per person tested and cost per HIV-positive individual identified were lower than the corresponding full cost estimates (Additional file 1 Table S5). Studies that used incremental costing methods likely underestimated costs as they did not include the health program’s existing infrastructure and overhead costs. These costs would potentially be incurred by those wishing to implement the same testing services in another setting where existing infrastructure many not be available. It is vital to consider the importance of the importance of financial vs economic costs in these settings since costs change as the epidemic changes and treatment strategies evolve. The financial cost is useful from the identified HIV program or organisation’s perspective. The economic cost is useful to capture the full value of the opportunity cost. Studies that estimated the financial costs might have costed a service that utilised donated goods or volunteer staff. The same service in another setting may have to purchase these goods or pay for staff. Using the GHCC’s principles (Table 4), our quality assessment found few studies fully accounted for donated goods and volunteer time.

We used the GHCC reference case to assess the quality of cost studies [35, 118] (Additional file 1 Table S4 & S5). The included cost components varied considerably. Though there has been a significant improvement in adherence to best practices for conducting and reporting findings from economic evaluations, the wide variability of unit costs is partly due to the non-standardised definition of unit cost and approaches to data collection and cost analysis reporting. Cost components and sources for cost data collection also varied, including estimating costs from a single health facility and aggregating data from all regions in a country without accounting for variations in HIV prevalence and population demographics.

### Limitations

This review has several limitations. We acknowledge the diversity and complexity of healthcare systems in sub-Saharan Africa. Thus, the review presented the cost studies’ results following the study perspective, not by implementation entity (such as government or partners). In no single country were all six HIV testing modalities assessed, making comparing different testing modalities difficult. No study reported cost per positive case identified for the key populations. The shadow price for goods and opportunity costs of time, characterising heterogeneity and uncertainty, could have been better reported. Thus, it took time to identify economic or financial costing methods accurately. The methods used to undertake the economic analysis were only sometimes comprehensive or comparable, limiting the generalisability of the findings. Moreover, we extracted data from diverse published sources, such as peer-reviewed papers, posters, abstracts, and presentations, limiting the quality assessment and comparison between studies. Some studies proposed checklists for the transferability of economic evaluations [119–122].

## Conclusion

Although the cost per person tested estimates varied widely, this study presented the costs of different HIV testing approaches for diverse populations and settings that would be informative for sub-Saharan Africa. We identified many studies reporting the incremental costs of different HIV testing modalities, but few studies undertook full costing.

### Supplementary Information

Additional file 1. Provides an overview of the papers included in this systematic literature review, including PICOS, Inclusion and exclusion criteria, PRISMA checklist, quality assessment, summary of incremental and full cost estimates and HTS cost studies included

## Data Availability

N/A.

## Abbreviations

$ppositive: Cost Per HIV-Positive Person Identified
$pptested: Cost Per Person Tested
ANC: Antenatal Clinic
GHCC: Global Health Cost Consortium
HIV: Human Immunodeficiency Virus
HTS: HIV Testing Services
IQR: Interquartile Range
OPD: Outpatient Department
PICOS: Population, Intervention, Comparators, Outcomes, And Study Type
PITC: Provider-Initiated HIV Counselling and Testing
PLHIV: People Living with HIV
PRISMA: Preferred Reporting Items for Systematic Reviews and Meta-Analysis
UNAIDS: The Joint United Nations Programme On HIV/AIDS
US$: U.S. Dollars
VMMC: Voluntary Male Medical Circumcision
VCT: Voluntary Counselling and Testing
