# Supplementary material for: Costs of HIV testing services in sub-Saharan Africa: a systematic literature review
Source: BMC Infect Dis. 2024 Aug 27;22(Suppl 1):980. doi: 10.1186/s12879-024-09770-7 (PMC11348535; doi:10.1186/s12879-024-09770-7)
Supplement: Supplementary file 1 — Additional file 1. Provides an overview of the papers included in this systematic literature review, including PICOS, Inclusion and exclusion criteria, PRISMA checklist, quality assessment, summary of incremental and full cost estimates and HTS cost studies included [file 12879_2024_9770_MOESM1_ESM.docx]

Additional file 1

**Title: Costs Analyses of HIV testing services in sub-Saharan Africa: a systematic literature review**

**Authors:** Nurilign Ahmed, Jason J. Ong, Kathleen McGee, Marc d’Elbée, Cheryl Johnson, Valentina Cambiano, Karin Hatzold, Elizabeth L Corbett, Fern Terris-Prestholt, Hendramoorthy Maheswaran

Additional files

Supplementary Tables

Table S1 PICOS Inclusion and exclusion criteria

| **PICOS** | **Inclusion criteria** | **Exclusion criteria** |
| --- | --- | --- |
| **Population** | Adolescents, adult men, and adult women | Infants and children (<age 16) |
| **Intervention** | Different types of HTS (differentiated HIV testing services) | Infant and children HIV testing approaches |
| **Comparators** | Any stated comparators | None |
| **Outcomes** | Cost estimates are cost per person tested and per HIV + person identified. | Not stating costs measures or units of health outcomes in the study |
| **Study types** | Costing and cost-effectiveness analysis of HTS in sub-Saharan Africa | Costing: where no new primary costs data are presented.  Cost-effectiveness: where no new primary costs data are presented. |

Table S2 Systematic literature review search strategy and strings

| Searched databases | Search terms | Result |
| --- | --- | --- |
| Medline | | |
| Concept 1(C1) | HIV Infections OR HIV OR hiv OR hiv-1 OR hiv-2 OR hiv1 OR hiv2 OR hiv infect* OR human immunodeficiency virus OR human immunedeficiency virus OR human immuno-deficiency virus OR human immune-deficiency virus OR ((human immun*) AND (deficiency virus)) OR acquired immunodeficiency syndrome OR acquired immunedeficiency syndrome OR acquired immuno-deficiency syndrome OR acquired immune-deficiency syndrome OR ((acquired immun*) AND (deficiency syndrome)) OR Sexually Transmitted Diseases | 211,320 |
| Concept 2(C2) | Counselling OR Counseling OR Counse*OR Testing OR Test* | 386,102 |
| Concept 3 (C3) | Cost OR Costs OR Costing OR Cost-effectiveness OR Cost-effectiveness analysis OR Cost effectiveness analysis OR Effec* OR effectives* OR Cost* | 1,800,445 |
| C1 AND C2 AND C3 |  | 461 |
| Concept 4 | hiv self-testing OR self-test* OR "self test" OR hiv self-test OR hivst OR home test* | 1,581 |
| PubMed* | | |
| C1 AND C2 AND C3 | HIV Infections OR HIV OR hiv OR hiv-1 OR hiv-2 OR hiv1 OR hiv2 OR hiv infect* OR human immunodeficiency virus OR human immunedeficiency virus OR human immuno-deficiency virus OR human immune-deficiency virus OR ((human immun*) AND (deficiency virus)) OR acquired immunodeficiency syndrome OR acquired immunedeficiency syndrome OR acquired immuno-deficiency syndrome OR acquired immune-deficiency syndrome OR ((acquired immun*) AND (deficiency syndrome)) OR Sexually Transmitted Diseases AND Counselling OR Counseling OR Counse* OR Testing OR Test* AND Cost OR Costs OR Costing OR Cost-effectiveness OR Cost-effectiveness analysis OR Cost effectiveness analysis OR Effec* OR effectives* OR Cost* | 980 |
| Concept 4 | hiv self-testing OR self-test* OR "self test" OR hiv self-test OR hivst OR home test* | 639 |
| EMBASE | | |
| Concept 1 | HIV Infections OR HIV OR hiv OR hiv-1 OR hiv-2 OR hiv1 OR hiv2 OR hiv infect$ OR human immunodeficiency virus OR human immunedeficiency virus OR human immuno-deficiency virus OR human immune-deficiency virus OR ((human immune$) AND (deficiency virus)) OR acquired immunodeficiency syndrome OR acquired immunedeficiency syndrome OR acquired immuno-deficiency syndrome OR acquired immune-deficiency syndrome OR ((acquired immune$) AND (deficiency syndrome)) OR Sexually Transmitted Diseases | 256,689 |
| Concept 2 | Counselling OR Counseling OR Counse*OR Testing OR Test* | 495,348 |
| Concept 3 | Cost OR Costs OR Costing OR Cost-effectiveness OR Cost-effectiveness analysis OR Cost effectiveness analysis OR Effec* OR effectives* OR Cost* | 2,320,362 |
| C1 AND C2 AND C3 |  | 569 |
| Concept 4 | hiv self-testing OR hiv self-test OR hivst OR home test* OR rapid test* | 1993 |
| Popline | | |
| C1 AND C2 AND C3 | HIV Infections* OR HIV OR human immunodeficiency virus* OR acquired immunodeficiency syndrome* OR AIDS **And** Counselling OR Counseling OR Counse*OR Testing OR Test* **AND** Cost OR Costing OR Cost-effectiveness OR Cost-effectiveness analysis OR Cost effectiveness analysis OR Effec* OR effectives* | 175 |
| Concept 4 | hiv self-test* OR hiv self-testing | 68 |
| SCOPUS* | | |
| C1 AND C2 AND C3 | HIV Infections OR HIV OR hiv OR hiv-1 OR hiv-2 OR hiv1 OR hiv2 OR hiv infect$ OR human immunodeficiency virus OR human immunedeficiency virus OR human immuno-deficiency virus OR human immune-deficiency virus OR ((human immune$) AND (deficiency virus)) OR acquired immunodeficiency syndrome OR acquired immunedeficiency syndrome OR acquired immuno-deficiency syndrome OR acquired immune-deficiency syndrome OR ((acquired immune$) AND (deficiency syndrome)) OR Sexually Transmitted Diseases AND Counselling OR Counseling OR Counse*OR Testing OR Test* AND Cost OR Costs OR Costing OR Cost-effectiveness OR Cost-effectiveness analysis OR Cost effectiveness analysis OR Effec* OR effectives* OR Cost* | 2,452 |
| Concept 4 | HIV* OR hiv self-testing OR hiv self-test* OR hivst OR home test* OR rapid test* | 1,536 |
| Global Health | | |
| Concept 1 | HIV Infections OR HIV OR hiv OR hiv-1 OR hiv-2 OR hiv1 OR hiv2 OR hiv infect* OR human immunodeficiency virus OR human immunedeficiency virus OR human immuno-deficiency virus OR human immune-deficiency virus OR ((human immun*) AND (deficiency virus)) OR acquired immunodeficiency syndrome OR acquired immunedeficiency syndrome OR acquired immuno-deficiency syndrome OR acquired immune-deficiency syndrome OR ((acquired immun*) AND (deficiency syndrome)) OR Sexually Transmitted Diseases | 110,964 |
| Concept 2 | Counselling OR Counseling OR Counse*OR Testing OR Test* | 62,706 |
| Concept 3 | Cost OR Costs OR Costing OR Cost-effectiveness OR Cost-effectiveness analysis OR Cost effectiveness analysis OR Effec* OR effectives* OR Cost* | 338,534 |
| C1 AND C2 AND C3 |  | 313 |
| Concept 4 | hiv self-testing OR self-test* OR "self test" OR hiv self-test OR hivst OR home test* | 972 |
| COCHRANE* | | |
| C1 AND C2 AND C3 | HIV Infections OR HIV OR hiv OR hiv-1 OR hiv-2 OR hiv1 OR hiv2 OR hiv infect* OR human immunodeficiency virus OR human immunedeficiency virus OR human immuno-deficiency virus OR human immune-deficiency virus OR ((human immun*) AND (deficiency virus)) OR acquired immunodeficiency syndrome OR acquired immunedeficiency syndrome OR acquired immuno-deficiency syndrome OR acquired immune-deficiency syndrome OR ((acquired immun*) AND (deficiency syndrome)) OR Sexually Transmitted Diseases AND Counselling OR Counseling OR Counse*OR Testing OR Test* AND Cost OR Costs OR Costing OR Cost-effectiveness OR Cost-effectiveness analysis OR Cost effectiveness analysis OR Effec* OR effectives* OR Cost* | 51 |
| Concept 4 | hiv self-testing OR self-test* OR "self test" OR hiv self-test OR hivst OR home test* | 0 |
| Social policy and practice | | |
| Concept 1 | HIV Infections OR HIV OR hiv OR hiv-1 OR hiv-2 OR hiv1 OR hiv2 OR hiv infect* OR human immunodeficiency virus OR human immunedeficiency virus OR human immuno-deficiency virus OR human immune-deficiency virus OR ((human immun*) AND (deficiency virus)) OR acquired immunodeficiency syndrome OR acquired immunedeficiency syndrome OR acquired immuno-deficiency syndrome OR acquired immune-deficiency syndrome OR ((acquired immun*) AND (deficiency syndrome)) OR Sexually Transmitted Diseases | 5,138 |
| Concept 2 | Counselling OR Counseling OR Counse*OR Testing OR Test* | 18,579 |
| Concept 3 | Cost OR Costs OR Costing OR Cost-effectiveness OR Cost-effectiveness analysis OR Cost effectiveness analysis OR Effec* OR effectives* OR Cost* | 83,039 |
| C1 AND C2 AND C3 |  | 161 |
| Concept 4 | hiv self-testing OR self-test* OR "self test" OR hiv self-test OR hivst OR home test* | 0 |
| Web of Science | | |
| C1 AND C2 AND C3 | HIV Infections OR HIV OR hiv OR hiv-1 OR hiv-2 OR hiv1 OR hiv2 OR hiv infect* OR human immunodeficiency virus OR human immunedeficiency virus OR human immuno-deficiency virus OR human immune-deficiency virus OR ((human immun*) AND (deficiency virus)) OR acquired immunodeficiency syndrome OR acquired immunedeficiency syndrome OR acquired immuno-deficiency syndrome OR acquired immune-deficiency syndrome OR ((acquired immun*) AND (deficiency syndrome)) OR Sexually Transmitted Diseases AND Counselling OR Counseling OR Counse*OR Testing OR Test* AND Cost OR Costs OR Costing OR Cost-effectiveness OR Cost-effectiveness analysis OR Cost effectiveness analysis OR Effec* OR effectives* OR Cost* | 513 |
| Concept 4 | hiv self-testing OR self-test* OR "self test" OR hiv self-test OR hivst OR home test* | 1,060 |
| Tuft's cost effectiveness analysis registry | HIV | 98 |

*Pubmed, SCOPUS, COCHRANE and Web of Science databases were searched using "AND" conjugation concepts 1, 2, and 3.

Table S3 PRISMA 2009 Checklist

| **Section/topic** | **#** | **Checklist item** | **Reported in section** |
| --- | --- | --- | --- |
| **TITLE** | | |  |
| Title | 1 | Identify the report as a systematic review, meta-analysis, or both. | Title section |
| **ABSTRACT** | | |  |
| Structured summary | 2 | Provide a structured summary including, as applicable: background; objectives; data sources; study eligibility criteria, participants, and interventions; study appraisal and synthesis methods; results; limitations; conclusions and implications of key findings; systematic review registration number. | Supplemental appendix |
| **INTRODUCTION** | | |  |
| Rationale | 3 | Describe the rationale for the review in the context of what is already known. | Introduction |
| Objectives | 4 | Provide an explicit statement of questions being addressed with reference to participants, interventions, comparisons, outcomes, and study design (PICOS). | Introduction |
| **METHODS** | | |  |
| Protocol and registration | 5 | Indicate if a review protocol exists, if and where it can be accessed (e.g., Web address), and, if available, provide registration information including registration number. | Systematic literature review not registered |
| Eligibility criteria | 6 | Specify study characteristics (e.g., PICOS, length of follow-up) and report characteristics (e.g., years considered, language, publication status) used as criteria for eligibility, giving rationale. | Methods and supplemental table |
| Information sources | 7 | Describe all information sources (e.g., databases with dates of coverage, contact with study authors to identify additional studies) in the search and date last searched. | Methods and supplemental table |
| Search | 8 | Present full electronic search strategy for at least one database, including any limits used, such that it could be repeated. | Supplemental table |
| Study selection | 9 | State the process for selecting studies (i.e., screening, eligibility, included in systematic review, and, if applicable, included in the meta-analysis). | Methods |
| Data collection process | 10 | Describe method of data extraction from reports (e.g., piloted forms, independently, in duplicate) and any processes for obtaining and confirming data from investigators. | Methods |
| Data items | 11 | List and define all variables for which data were sought (e.g., PICOS, funding sources) and any assumptions and simplifications made. | Methods |
| Risk of bias in individual studies | 12 | Describe methods used for assessing risk of bias of individual studies (including specification of whether this was done at the study or outcome level), and how this information is to be used in any data synthesis. | Methods |
| Summary measures | 13 | State the principal summary measures (e.g., risk ratio, difference in means). | Methods |
| Synthesis of results | 14 | Describe the methods of handling data and combining results of studies, if done, including measures of consistency (e.g., I^2^) for each meta-analysis. | Methods and supplemental table |
| Risk of bias across studies | 15 | Specify any assessment of risk of bias that may affect the cumulative evidence (e.g., publication bias, selective reporting within studies). | Discussion |
| Additional analyses | 16 | Describe methods of additional analyses (e.g., sensitivity or subgroup analyses, meta-regression), if done, indicating which were pre-specified. | Supplemental table |

| **RESULTS** | | |  |
| --- | --- | --- | --- |
| Study selection | 17 | Give numbers of studies screened, assessed for eligibility, and included in the review, with reasons for exclusions at each stage, ideally with a flow diagram. | Results and supplemental table |
| Study characteristics | 18 | For each study, present characteristics for which data were extracted (e.g., study size, PICOS, follow-up period) and provide the citations. | Results and supplemental table |
| Risk of bias within studies | 19 | Present data on risk of bias of each study and, if available, any outcome level assessment (see item 12). |  |
| Results of individual studies | 20 | For all outcomes considered (benefits or harms), present, for each study: (a) simple summary data for each intervention group (b) effect estimates and confidence intervals, ideally with a forest plot. | Results and supplemental table |
| Synthesis of results | 21 | Present results of each meta-analysis done, including confidence intervals and measures of consistency. | Meta-analysis not done |
| Risk of bias across studies | 22 | Present results of any assessment of risk of bias across studies (see Item 15). | Discussion |
| Additional analysis | 23 | Give results of additional analyses, if done (e.g., sensitivity or subgroup analyses, meta-regression- see Item 16). | Results |
| **DISCUSSION** | | |  |
| Summary of evidence | 24 | Summarise the main findings including the strength of evidence for each main outcome; consider their relevance to key groups (e.g., healthcare providers, users, and policy makers). | Discussion |
| Limitations | 25 | Discuss limitations at study and outcome level (e.g., risk of bias), and at review-level (e.g., incomplete retrieval of identified research, reporting bias). | Discussion |
| Conclusions | 26 | Provide a general interpretation of the results in the context of other evidence, and implications for future research. | Discussion |
| **FUNDING** | | |  |
| Funding | 27 | Describe sources of funding for the systematic review and other support (e.g., supply of data), role of funders for the systematic review. | Funding statement |

*From:* Moher D, Liberati A, Tetzlaff J, Altman DG, The PRISMA Group (2009). Preferred Reporting Items for Systematic Reviews and Meta-Analyses: The PRISMA Statement. PLoS Med 6(6): e1000097. doi:10.1371/journal.pmed1000097 For more information, visit: **www.prisma-statement.org**

Table S4 Quality assessment using the GHCC's principles and methods reporting checklist for cost studies [[109](#_ENREF_109)]

| **Principle** | **Item No** | **GHCC reference case checklist items included** |
| --- | --- | --- |
| Principle 1 | P1 | The purpose of the study, the population, and the intervention and/or service/output being costed should be clearly defined. |
| Principle 2 | P2 | The perspective (extent of the resource use captured) of the cost estimation should be stated and justified relevant to purpose. |
| Principle 3 | P3 | The type of cost being estimated should be clearly defined, regarding economic vs. financial, real-world vs. guideline, and incremental vs. full cost, and whether the cost is 'net of future cost,' should be justified relevant to purpose. |
| Principle 4 | P4 | The 'units' in the unit costs for strategies, services, and interventions should be defined, relevant for the costing purpose, and generalisable. |
| Principle 5 | P5 | The time horizon should be of sufficient length to capture all costs relevant to the purpose, and consideration should be given to disaggregating costs into separate periods where appropriate. |
| Principle 6 | P6 | The scope of the inputs to include in the cost estimation should be defined and justified relevant to purpose. |
| Principle 7 | P7 | The methods for estimating the number of inputs should be described, including data sources and criteria for allocating resources (Describe the measurement of each input as either top-down or bottom-up, a method to allocate human resources inputs, overhead and other resources and methods for excluding research costs). |
| Principle 8 | P8 | The sampling strategy used should be determined by the precision demanded by the costing purpose and designed to minimise. |
| Principle 9 | P9 | The selection of the data source(s) and methods for estimating service use should be described, and potential biases reported in the study limitations. |
| Principle 10 | P10 | Consideration should be given to the timing of data collection to minimise recall bias and, where relevant, the impact of seasonality and other differences over time. |
| Principle 11 | P11 | The sources for price data should be listed by input, and clear delineation should be made between local and international price data sources, and tradeable, non-tradeable goods (Report the sources of price data by input and where local and international prices were uses). |
| Principle 12 | P12 | Capital costs should be appropriately annuitized or depreciated to reflect the expected life of capital inputs (Describe the depreciation approach, discount rate used from capital goods, and expected life years of capital goods and data source). |
| Principle 13 | P13 | Where relevant an appropriate discount rate, inflation and exchange rates should be used, and clearly stated (discount rate used for future costs, currency year, conversion made and inflation type, and rate used). |
| Principle 14 | P14 | The use and source of shadow prices for goods and for the opportunity cost of time should be reported (Report methods for valuing volunteer time and adjustments for input prices for donated or subsidised goods). |
| Principle 15 | P15 | Variation in the cost of the intervention by site size/organisation, sub-populations, or by other drivers of heterogeneity should be explored and reported. |
| Principle 16 | P16 | The uncertainty associated with cost estimates should be appropriately characterised (describe sensitivity analyses conducted and list of possible sources of bias). |
| Principle 17 | P17 | Cost estimates should be communicated clearly and transparently to enable decision-maker(s) to interpret and use the results (limitations, conflicts of interest and open access). |

**Table S5 Summary of incremental and full cost estimates in 2019 USD**

|  | Mean Incremental Costs | | Mean Full Costs | |
| --- | --- | --- | --- | --- |
| Cost estimates (*n*) | **$pptested (Median IQR) (*n*)** | **$ppositive (Median IQR) (*n*)** | **$pptested (Median IQR) (*n*)** | **$ppositive (Median IQR) (*n*)** |
| Total (*n*=167) | $18.45 (median=$12.26, IQR: $7.64-$23.50) (*n*=124) | $359.76 (median = $168.80, IQR: $80.08-$403.74) (*n*=71) | $38.65 (median=$32.83, IQR: $25.47-$45.69) (*n*=33) | $367.43 (median=$322.92, IQR: $85.22-$582.91) (*n*=16) |
|  |  |  |  |  |
| Countries (*n*) | | | | |
| Botswana *(n=2)* | - | - | $44.40 (median=$44.40, IQR: $39.55-$49.25) (*n*=2) | $678.77 (median=$678.77, IQR: $631.31-$726.24) (*n*=2) |
| Eswatini *(n=5)* | $11.58 (median=$9.33, IQR: $8.75-$12.16) (*n*=4) | $205.48 (median=$165.07, IQR: $48.33-$322.21) (*n*=4) | $31.13 (median=$31.13, IQR: $31.13-$) (*n*=1) | $121.42 (median=$121.42, IQR: $121.42 -$121.42) (*n*=1) |
| Ethiopia *(n=2)* | $31.53 (median=$31.53, IQR: $22.44-$40.16) (*n*=2) | - | - | - |
| Kenya *(n=29)* | $25.11 (median=$15.64, IQR: $12.78-$37.33) (*n*=15) | $178.56 (median=$116.80, IQR: $66.84-$168.80) (*n*=9) | $32.77 (median=$26.89, IQR: $15.95-$45.39) (*n*=10) | $139.41 (median=$53.58, IQR: $30.42-$268.54) (*n*=5) |
| Lesotho *(n=13)* | $14.11 (median=$14.08, IQR: $13.72-$14.46) (*n*=4) | $300.13 (median=$300.13, IQR: $253.42-$346.84) (*n*=2) | $29.33 (median=$32.20, IQR: $23.50-$35.00) (*n*=9) | $670.08 (median=$670.08, IQR: $496.42-$843.74) (*n*=2) |
| Malawi *(n=20)* | $13.03 (median=$9.82 IQR: $6.25-$12.84) (*n*=19) | $149.00 (median=$121.64, IQR: $96.84-$169.16) (*n*=11) | #DIV/--0! | #DIV-/-0! |
| Namibia *(n=3)* | - | - | $48.03 (median=$60.28, IQR: $40.67-$61.51) (*n*=3) | $587.80 (median=$587.80, IQR: $582.35-$593.24) (*n*=2) |
| Nigeria *(n=10)* | $22.55 (median = $16.96, IQR: $9.19-$34.09) (*n*=8) | $1,297.86 (median=$931.18, IQR: $444.57-$1,784.47) (*n*=4) | - | - |
| Rwanda *(n=3)* | $10.09 (median=$9.51, IQR: $7.01-$12.87) (*n*=3) | $1,528.00 (median=$1528.00, IQR: $1,380.52-$1675.48) (*n*=2) | - | - |
| South Africa *(n=23)* | $25.91 (median=$13.38, IQR: $7.38-$29.96) (*n*=22) | $409.78 (median=$156.45, IQR: $19.01-$723.11) (*n*=13) | - | - |
| Tanzania *(n=5)* | $8.30 (median=$6.73, IQR: $5.73-$8.32) (*n*=5) | $353.08 (median=$379.20, IQR: $309.51-$422.76) (*n*=4) | - | - |
| Uganda *(n=31)* | $13.76 (median=$10.95, IQR: $6.43-$15.64) (*n*=26) | $226.26 (median=$148.40, IQR: $82.10-$246.75) (*n*=13) | $33.56 (median=$31.20, IQR: $30.75-$35.19) (*n*=3) | $522.45 (median=$522.45, IQR: $492.38-$552.53) (*n*=2) |
| Zambia *(n=16)* | $21.08 (median=$14.07, IQR: $7.13-$26.43) (*n*=14) | $345.71 (median=$390.39, IQR: $85.43-$522.94) (*n*=8) | $59.73 (median=$59.63, IQR: $32.38-$86.99) (*n*=4) | $71.14 (median=$71.14, IQR: $58.83-$83.45) (*n*=2) |
| Zimbabwe *(n=3)* | $11.35 (median=$11.35, IQR: $10.11-$12.60) (*n*=2) | $180.55 (median=$180.55, IQR: $180.55-$180.55) (*n*=1) | $80.00 (median=$80.00, IQR: $80.00 -$80.00) (*n*=1) | - |
| Other Western AU countries (*n*=10) | $22.55 (median=$16.96, IQR: $9.19-$34.09) (*n*=8) | $1,297.86 (median=$931.18, IQR: $444.57-$1,784.47) (*n*=4) |  |  |
| Other Southern AU countries (*n*=85) | $19.05 (median=$12.13, IQR: $7.29-$19.68) (*n*=65) | $290.63 (median=$156.45, IQR: $72.26-$403.74) (*n*=39) | $42.35 (median=$34.50, IQR: $30.38-$55.64) (*n*=20) | $459.67 (median=$576.91, IQR: $121.42-$598.69) (*n*=9) |
| Other Eastern AU countries (*n*=70) | $20.28 (median=$12.44, IQR: $8.11-$18.84) (*n*=51) | $322.03 (median=$161.60, IQR: $85.38-$339.46) (*n*=32) | $32.95 (median=$30.30, IQR: $16.08-$44.47) (*n*=14) | $248.85 (median=$268.54, IQR: $42.00-$392.69) (*n*=5) |
|  |  |  |  |  |
| Income Level | | | | |
| Low income *(n=56)* | $13.97 (median=$10.43, IQR: $6.18-$15.42) (*n*=50) | $293.71 (median=$144.27, IQR: $92.70-$241.97) (*n*=26) | $ 33.56 (median=$31.20, IQR: $30.70-$35.19) (*n*=3) | $522.45 (median=$522.45, IQR: $492.38-$522.53) (*n*=2) |
| Lower-middle income *(n=81)* | $19.60 (median=$13.96, IQR: $8.31-$25.76) (*n*=53) | $393.10 (median=$243.97, IQR: $88.63-$452.96) (*n*=31) | $37.10 (median=$32.20, IQR: $23.50-$44.47) (*n*=24) | $230.09 (median=$108.59, IQR: $48.28-$309.20) (*n*=9) |
| Upper-middle income *(n=28)* | $25.91 (median=$13.38, IQR: $7.38-$29.96) (*n*=19) | $409.78 (median=$156.45, IQR: $19.01$723.11) (*n*=11) | $46.58 (median=$54.10, IQR: $34.70-$60.28) (*n*=4) | $633.29 (median=$591.27, IQR: $582.11-$642.44) (*n*=5) |
|  |  |  |  |  |
| Country HIV prevalence (year of costing*)* | | | | |
| 1%-5% *(n=15)* | $21.05 (median=$13.36, IQR: $9.49-$30.49) (*n*=13) | $1,374.57 (median=$1,309.58, IQR: $665.45-$1,713.74) (*n*=6) | - | - |
| 5%-10% *(n=71)* | $15.59 (median=$10.95, IQR: $6.39-$16.27) (*n*=52) | $217.52 (median=$148.40, IQR: $95.79-$237.19) (*n*=31) | $32.95 (median=$30.30, IQR: $16.08-$44.47) (*n*=13) | $248.85 (median=$268.54, IQR: $42.00-$392.69) (*n*=7) |
| 10%-15% *(n=41)* | $17.38 (median=$12.79, IQR: $7.93-$15.83) (*n*=34) | $225.66 (median=$113.04, IQR: $73.66-$393.67) (*n*=17) | $57.88 (median=$61.51, IQR: $32.38-$81.61) (*n*=8) | $329.47 (median=$336.34, IQR: $83.45-$582.35) (*n*=4) |
| 15%-20% *(n=18)* | $29.99 (median=$23.35, IQR: $7.08-$48.85) (*n*=17) | $48125 (median=$356.22, IQR: $22.24-$864.86) (*n*=11) | - | - |
| 20%-25% *(n=15)* | $14.11 (median=$14.08, IQR: $13.72-$14.46) (*n*=4) | $300.13 (median=$300.13, IQR: $253.42-$346.84) (*n*=2) | $32.07 (median=$34.30, IQR: $26.00-$36.35) (*n*=11) | $674.43 (median=$678.77, IQR: $518.57-$834.63) (*n*=4) |
| 25%-30% *(n=5)* | $11.58 (median=$9.33, IQR: $8.75-$12.16) (*n*=4) | $205.48 (median=$165.07, IQR: $48.33-$322.21) (*n*=4) | $31.13 (median=$31.13, IQR: $31.13 -$31.13) (*n*=1) | $121.42 (median=$121.42, IQR: $121.42 -$121.42) (*n*=1) |
| Cost Year | | | | |
| 2000-2005 (*n*=5) | $15.28 (median=$15.51, IQR: $14.22-$16.57) (*n*=4) |  | $39.18 (median=$39.18, IQR: $39.18-$39.18) (*n*=1) | - |
| 2005-2010 (*n*=36) | $16.34 (median=$11.77, IQR: $8.15-$15.12) (*n*=27) | $116.91 (median=$94.62, IQR: $48.33-$152.09) (*n*=16) | $44.04 (median=$44.47, IQR: $29.67-$59.10) (*n*=11) | $318.25 (median=$295.81, IQR: $138.96-$513.45) (*n*=6) |
| 2010-2015(*n*=71) | $20.32 (median=$14.03, IQR: $8.23-$28.63) (*n*=60) | $465.55 (median=$203.97, IQR: $86.47-$512.75) (*n*=33) | $55.57 (median=$58.78, IQR: $27.37-$86.99) (*n*=4) | $56.71 (median=$42.00, IQR: $28.17-$70.54) (*n*=3) |
| 2015-2020 (*n*=53) | $17.16 (median=$10.08, IQR: $5.73-$14.23) (*n*=34) | $377.69 (median=$352.88, IQR: $131.77-$506.29) (*n*=19) | $31.14 (median=$31.20, IQR: $19.27-$35.00) (*n*=18) | $623.77 (median=$583.22, IQR: $492.38-$726.24) (*n*=6) |
|  |  |  |  |  |
| HTS Modality | | | | |
| Campaign style *(n=13)* | $27.64 (median=$26.70, IQR: $12.42-$41.93) (*n*=4) | $413.14 (median=$388.70, IQR: $258.16-$555.91) (*n*=3) | $36.88 (median=$34.65, IQR: $31.28-$40.50) (*n*=8) | - |
| Facility bas  ed *(n=72)* | $19.63 (median=$10.70, IQR: $6.00-$28.63) (*n*=56) | $398.95 (median=$148.29, IQR: $69.85-$429.42) (*n*=32) | $44.03 (median=$31.20, IQR: $26.04-$70.14) (*n*=11) | $191.08 (median=$74.67, IQR: $42.49-$235.29) (*n*=8) |
| Facility - ANC/PMTCT *(n=13)* | $42.74 (median=$46.75, IQR: $16.24-$66.62) (*n*=9) | $967.23 (median=$518.84, IQR: $399.42-$1,039.32) (*n*=8) | $44.09 (median=$31.20, IQR: $12.42-$41.93) (*n*=3) | $582.60 (median=$26.70, IQR: $12.42-$41.93) (*n*=1) |
| Facility - VCT stand-alone *(n=34)* | $14.68 (median=$10.71, IQR: $6.18-$16.02) (*n*=26) | $276.35 (median=$122.62, IQR: $72.96-$171.74) (*n*=16) | $48.91 (median=$32.83, IQR: $31.01-$86.44) (*n*=5) | $48.53 (median=$38.47, IQR: $28.17-$58.83) (*n*=4) |
| Facility – Integrated *(n=10)* | $33.77 (median=$18.40, IQR: $14.21-$47.91) (*n*=7) | $19.31 (median=$19.31, IQR: $19.31-$19.31) (*n*=3) | $23.34 (median=$23.61, IQR: $19.84-$27.34) (*n*=2) | $87.50 (median=$87.50, IQR: $70.54-$104.46) (*n*=2) |
| Facility – OPD *(n=15)* | $6.91 (median=$6.53, IQR: $3.14-$8.09) (*n*=13) | $83.96 (median=$66.84, IQR: $35.45-$125.31) (*n*=5) | $60.28 (median=$60.28, IQR: $60.28-$60.28) (*n*=1) | $576.91 (median=$576.91, IQR: $576.91 -$576.91) (*n*=1) |
| Home based *(n=32)* | $19.30 (median=$13.42, IQR: $8.34-$23.35) (*n*=29) | $297.09 (median=$246.75, IQR: $132.60-$381.62) (*n*=15) | $27.83 (median=$19.27, IQR: $14.69-$36.68) (*n*=3) | $704.62 (median=$773.70, IQR: $548.23-$895.55) (*n*=3) |
| Mobile *(n=18)* | $16.47 (median=$12.88, IQR: $9.88-$23.94) (*n*=13) | $356.93 (median = $206.71, IQR: $126.32-$387.29) (*n*=11) | $37.81 (median=$31.51, IQR: $27.61-$41.71) (*n*=4) | $483.69 (median=$583.85, IQR: $426.19-$591.27) (*n*=3) |
| Self-testing *(n=25)* | $12.75 (median=$11.50, IQR: $9.27-$13.92) (*n*=19) | $338.57 (median=$113.04, IQR: $78.06-$516.30) (*n*=9) | $28.18 (median=$30.30, IQR: $15.90-$37.70) (*n*=5) | $462.30 (median=$462.30, IQR: $462.30-$462.30) (*n*=1) |
| ST- Community *(n=8)* | $9.83 (median=$9.84, IQR: $5.48-$14.17) (*n*=6) | $529.59 (median=$529.59, IQR: $522.94-$536.23) (*n*=2) | $40.55 (median=$40.55, IQR: $39.13-$41.98) (*n*=2) | - |
| ST- Facility *(n=12)* | $10.70 (median=$10.55, IQR: $10.18-$12.25) (*n*=9) | $92.00 (median=$83.32, IQR: $44.12-$106.92) (*n*=6) | $14.75 (median=$14.75, IQR: $14.17-$15.32) (*n*=2) | - |
| ST- Home *(n=5)* | $21.76 (median=$14.03, IQR: $12.83-$22.96) (*n*=4) | $1,435.94 (median=$1,435.94, IQR: $1,435.94-$1,435.94) (*n*=1) | $30.30 (median=$30.30, IQR: $30.30 -$30.30) (*n*=1) | $462.30 (median=$462.30, IQR: $462.30 -$462.30) (*n*=1) |
| Stand-alone *(n=2)* | $20.61 (median=$20.52, IQR: $15.10-$26.08) (*n*=3) | $107.15 (median=$107.15, IQR: $107.15-$107.15) (*n*=1) | $60.16 (median=$60.16, IQR: $52.93 -$67.40) (*n*=2) | $323.08 (median=$323.08, IQR: $323.08 -$323.08) (*n*=1) |
|  |  |  |  |  |
| Primary vs Secondary Testing | | | | |
| Primary/Direct testing *(n=140)* | $16.71 (median=$10.95, IQR: $7.24-$18.72) (*n*=104) | $340.16 (median=$161.60, IQR: $79.07-$393.64) (*n*=66) | $39.16 (median=$34.30, IQR: $24.48 -$49.90) (*n*=31) | $398.52 (median=$322.92, IQR: $115.01 -$587.56) (*n*=12) |
| Secondary/Index^a^ testing *(n=25)* | $27.52 (median=$15.85, IQR: $14.41-$38.88) (*n*=20) | $618.48 (median=$356.22, IQR: $246.75-$1,041.58) (*n*=5) | $30.75 (median=$30.75, IQR: $30.53 -$30.98) (*n*=2) | $274.19 (median=$246, IQR: $28.17 -$492.38) (*n*=4) |
|  |  |  |  |  |
| Testing population | | | | |
| General population(s)^b^ (*n* = 97) | $14.39 (median=$10.25, IQR: $7.00-$15.52) (*n*=92) | $255.40 (median=$148.40, IQR: $72.26-$348.18) (*n*=59) | - | - |
| PLHIV Partners (*n* = 16) | $19.31 (median=$15.57, IQR: $14.86-$27.09) (*n*=14) | $246.75 (median=$246.75, IQR: $246.75-$246.75) (*n*=1) | - | $25.92 (median=$25.92, IQR: $23.67-$28.17) (*n*=2) |
| ANC/PMTCT Male Partners (*n* = 8) | $47.94 (median=$49.17, IQR: $13.39-$55.19) (*n*=5) | $711.41 (median=$698.90, IQR: $270.14-$1,140.17) (*n*=4) | $30.75 (median=$30.75, IQR: $30.53-$30.98) (*n*=2) | $522.45 (median=$522.45, IQR: $492.38-$552.53) (*n*=2) |
| Pregnant women or women breastfeeding (*n* = 13) | $39.25 (median=$41.32, IQR: $14.08-$62.39) (*n*=10) | $1,054.52 (median=$524, IQR: $463.28-$1,300.53) (*n*=7) | $50.53 (median=$50.53, IQR: $35.80-$65.27) (*n*=2) | - |
| Key Population(s)^c^ (*n* = 6) | $20.31 (median=$9.49, IQR: $8.00-$27.21) (*n*=3) | - | $11.71 (median=$13.59, IQR: $9.62-$14.75) (*n*=3) | - |
|  |  |  |  |  |
| Type of Cost Analysis | | | | |
| Incremental (*n*=131) | $18.45 (median=$12.26, IQR: $7.64-$23.50) (*n*=124) | $359.76 (median=$168.80, IQR: $80.08-$403.73) (*n*=71) | - | - |
| Full (*n*=34) | - | - | $38.65 (median=$32.83, IQR: $25.47-$45.69) (*n*=33) | $367.43 (median=$322.92, IQR: $85.22-$582.91) (*n*=16) |
| Financial (*n*=75) | $19.13 (median=$13.11, IQR: $7.52-$12.88) (*n*=72) | $334.37 (median=$237.19, IQR: $79.28-$449.47) (*n*=40) | - | - |
| Economic (*n*= 90) | $25.71(median=$15.97, IQR: $9.82-$35.00) (*n*=85) | $383.98 (median=$157.03, IQR: $82.27-$494.50) (*n*=47) | - | - |
| Empirical (*n*=159) | $22.96 (median=$14.49, IQR: $8.76-$31.53) (*n*=154) | $363.93 (median=$177.58, IQR: $81.09-$474.91) (*n*=84) | - | - |
|  | $9.01 (median=$7.60, IQR: $5.49-$11.82) (*n*=3) | $283.75 (median=$349.54, IQR: $187.50-$412.89) (*n*=3) | - | - |

^a^ Secondary index testing focused on testing sexual partner(s) of HIV-positive individuals.

^b^ General population represented those people considered at risk of HIV acquisition and therefore deserving of HIV testing.

^c^ No study reported cost per positive case identified for key populations. “UNAIDS considers gay men and other men who have sex with men, sex workers, transgender people, people who inject drugs and prisoners and other incarcerated people as the five main key population groups that are particularly vulnerable to HIV and frequently lack adequate access to services.” Male truckers would fall into UNAID’s definition of vulnerable populations but not key populations [[41](#_ENREF_41)].

Table S6 Summary of HTS cost studies included 2006-2019 in 2019 USD (N=65)

| **Author, year, ref** | **Country** | **HTS**  **approach** | **Population** | **Costing method^1^** | **($pptested)** | **($ppositive)** | **Number of HIV tests provided** | **Number of HIV+ cases identified** | **Explicitly named cost inputs^2^** |
| --- | --- | --- | --- | --- | --- | --- | --- | --- | --- |
| Adebajo, 2013[[46](#_ENREF_46)] | Nigeria | Health facility, HTC | Key populations^a^ | Inc/Fin/Emp | $44.92 | - | 1,988 | 177 | NS |
|  |  | Mobile service, referred |  |  | $9.49 | - | 14,726 | 480 |  |
|  |  | Mobile service peer-lead |  |  | $6.51 | - | 14,895 | 1,853 |  |
| Ahmed, 2018[[43](#_ENREF_43)] | Zambia | Self-testing, Facility-based | General population | Inc/Fin/Emp | $13.34 | - | 12,885 | NA | TRNG, SNST BLDG, STOR, EQP, SUPL, VEH PER, TEST, REC |
|  |  | Self-testing, VMMC |  |  |  |  |  |  |  |
|  |  |  |  |  | $11.50 | - | 11,330 | NA |  |
|  |  | Self-testing, community- based |  |  |  |  |  |  |  |
|  |  |  |  |  | $14.23 | - | 103,589 | NA |  |
| Aliyu, 2012[[84](#_ENREF_84)] | Nigeria | Health facility | General population | Inc/Fin/Emp | $9.69 | - | NS | NA | TRNG, FURN EQP, PER, EST, ARV |
|  |  |  |  |  | $24.23 | - | NS | NA |  |
|  |  |  |  |  | $8.28 | - | NS | NA |  |
| Allen, 2014[[67](#_ENREF_67)] | Zambia | Health facility (CHCT) | General population | Inc/Fin/Emp | $40.28 | - | 148,839 | NA | TRNG, SNST, EQP, SUPL, VEH, PER, OVHD ADMN, M&E, TEST |
| Bassett, 2007[[68](#_ENREF_68)] | South Africa | Health facility OPD | General population | Inc/Fin/Emp | $7.29 | $21.98 | 137 | 102 | BLDG, PER, TEST |
|  |  |  |  |  | $7.66 | $11.47 | 1,414 | 463 |  |
| Bassett, 2014[[47](#_ENREF_47)] | South Africa | Mobile service | General population | Inc/Fin/Mod | - | $25.46 | 18,870 | 939 | VEH, PER |
| Bautista-Arredondo, 2016[[69](#_ENREF_69)] | Kenya | Health facility HTC | General population | Inc/Eco/Emp | $8.09 | $168.80 | 1,270 | 491 | TRNG, PER, SUPV |
|  |  | Health facility ANC/PMTCT | Pregnant women, or women breastfeeding |  | $68.21 | $778.11 | 288 | 105 |  |
|  | Rwanda | Health facility | General population |  | $4.51 | $1233.10 | 2,340 | 106 |  |
|  |  |  | Pregnant women, or women breastfeeding |  | $16.24 | $1823.04 | 812 | 14 |  |
|  | South Africa | Health facility | General population |  | $28.03 | $156.45 | 808 | 1,019 |  |
|  |  |  | Pregnant women, or women breastfeeding |  | $80.48 | $512.75 | 426 | 172 |  |
|  | Zambia | Health facility | General population |  | $13.92 | $89.35 | 242 | 291 |  |
|  |  |  | Pregnant women, or women breastfeeding |  | $35.89 | $413.81 | 618 | 104 |  |
| Bautista-Arredondo, (2018) [[110](#_ENREF_110)] | Nigeria | Health facility | General population | Inc/Eco/Emp | $30.49 | $1,386.12 | 141 | 139 | TRNG, EQP. VEH PER, SUPV |
|  |  |  | Pregnant women, or women breastfeeding |  | $46.75 | $2,979.54 | 137 | 131 |  |
| Bogart, 2017 [[85](#_ENREF_85)] | Uganda | Home-based | General population | Inc/Eco/Emp | $37.63 | - | 822 | 82 | TNSP, PER, TEST |
|  |  | Campaign style |  |  | $39.62 | - | 344 | 41 |  |
| Bulterys, (2020)[[106](#_ENREF_106)] | Uganda | Self-testing Facility-based PWLHW | ANC Male partners | Inc/Eco/Emp | $13.39 | - | - | - | TRNG SNST, BLDG, EQP SUPL, TNSP PER, OVHD, TEST, WST, REC |
|  |  | Self-testing Facility-based positive partner test |  |  | - | $11.89 | NS | NA |  |
|  |  | Self-testing Facility-based negative partner test |  |  | $10.55 | - | NS | NA |  |
| Cham, (2019)[[86](#_ENREF_86)] | Tanzania | Health facility OPD | General population | Inc/Eco/Emp | $4.75 | $128.98 | 88,813. | 3,270 | TRNG, EQP SUPL, TNSP, PER |
|  |  | Home-based |  |  | $6.73 | $369.69 | 27,407 | 499 |  |
|  |  | Campaign style |  |  | $8.32 | $388.70 | 17,475 | 374 |  |
| Change, 2016[[48](#_ENREF_48)] | Uganda (West) | Mobile service | General population | Inc/Eco/Emp | $11.22 | $166.17 | 4,417 | 287 | BLDG, EQP SUPL, PER |
|  |  |  |  |  | $24.36 | $288.84 | 771 | 57 |  |
|  | Uganda (East) | Mobile service | General population |  | $12.27 | $329.38 | 4,260 | 153 |  |
|  |  |  |  |  | $27.75 | $1,160.67 | 675 | 14 |  |
|  | Kenya | Mobile service | General population |  | $15.46 | $86.47 | 2,969 | 519 |  |
|  |  |  |  |  | $36.22 | $203.97 | 832 | 136 |  |
| Cherutich, (2018) [[107](#_ENREF_107)] | Kenya | Health facility - index | PLHIV Partners | Ful/Eco/Emp | - | $30.42 | NA | NS | BLDG, EQP SUPL, TNSP, OVHD, M&E, STRT |
|  |  | Health facility - index |  |  | - | $21.43 | NA | NS |  |
| DeBeer, (2015) **[**[111](#_ENREF_111)**]** | Namibia | Health facility | General population | Ful/Eco/Emp | $ 60.28 | $576.91 | 70,143. | 7,329 | TRNG, BLDG, FURN, TNSP, PER, TEST |
|  |  | Mobile service |  |  | $62.75 | 5$98.69 | 5,124. | 537 |  |
| d'Elbée,(2020) [87] | Lesotho | Campaign style | General population | Ful/Eco/Emp | $32.20 | - | 25,433 | NA | TRNG, SNST BLDG, STOR EQP ,SUPL ,TNSP ,VEH ,PER ,WST STRT |
|  |  | Campaign style |  |  | $28.50 | - | 27,045 | NA |  |
|  |  | Self-testing community-based |  |  | $43.40 | - | 6,300 | NA |  |
|  |  | Campaign style |  |  | $23.50 | - | 27,780 | NA |  |
|  |  | Self-testing community- based |  |  | $37.70 | - | 12,687 | NA |  |
|  |  | Campaign style |  |  | $35.00 | - | 27,045 | NA |  |
|  |  | Self-testing community-based |  |  | $15.40 | - | 6,300 | NA |  |
|  |  | Campaign style |  |  | $34.30 | - | 27,780 | NA |  |
|  |  | Self-testing community-based |  |  | $14.00 | - | 12,687 | NA |  |
| George, (2018) [[88](#_ENREF_88)] | Kenya | Self-testing | Key populations^b^ | Ful/Eco/Emp | $15.90 | - | NA | NA | TRNG, BLDG, EQP, SUPL, COMM, PER OVHD, SUPV |
|  |  | Self-testing |  |  | $13.59 | - | NA | NA |  |
|  |  | Health facility |  |  | $5.64 | - | NS | NA |  |
| Golovaty, (2018) [[89](#_ENREF_89)] | South Africa | Home-based | General population | Inc/Eco/Emp | $10.08 | - | 570.00 | NA | TRNG, MTG BLDG, EQP SUPL, COMM TNSP, PER OVHD, M&E TEST, STRT |
| Grabbe, (2010**) [**[49](#_ENREF_49)**]** | Kenya | Mobile service | General population | Ful/Eco/Emp | $25.47 | $268.54 | 47,539 | 4,265 | BLDG, EQP SUPL, VEH. PER OVHD |
|  |  | Mobile service |  |  | $28.32 | - | 41,829 | 3,782 |  |
|  |  | Stand-alone |  |  | $45.69 | $323.08 | 14,634 | 2,063 |  |
|  |  | Stand-alone |  |  | $74.63 | - | 8,415 | 1,612 |  |
| Hauck, 2018[[57](#_ENREF_57)] | Zambia | Home-based | General population | Inc/Fin/Emp | $26.77 | $366.97 | 126,208 | 9,196 | EQP, SUPL, TNSP, PER, ADMN |
|  |  |  |  |  | $25.42 | $691.82 | 136,966 | 4,921 |  |
| Hausler, 2006[[70](#_ENREF_70)] | South Africa | Health facility | General population | Ful/Eco/Emp | $15.05 | - | NS | NA | TRNG, BLDG, FURN EQP, VEH, PER ,SUPV ,TEST |
|  |  |  |  |  | $18.40 | - | NS | NA |  |
|  |  |  |  |  | $11.71 | - | NS | NA |  |
| Helleringer, 2013[[58](#_ENREF_58)] | Malawi | Home-based | General population | Inc/Fin/Emp | $12.13 | $150.45 | 597 | 48 | TRNG, SNST, TNSP |
|  |  |  |  |  | $13.42 | $393.67 | 586 | 45 |  |
| Hewett, (2016) [[90](#_ENREF_90)] | Zambia | Health facility | General population | Ful/Eco/Emp | $88.63 | - | NA | NA | NA |
|  |  |  |  |  | $86.44 | - | NA | NA |  |
|  |  |  |  |  | $82.04 | - | NA | NA |  |
| Ibekwe, 2017[[71](#_ENREF_71)] | Nigeria | Health facility | General population | Inc/Eco/Mod | - | $476.26 | NA | 15 | N.R. |
|  |  |  | Pregnant women or women breastfeeding |  | - | $349.54 | NA | 44 |  |
| Kabami ,(2017) **[**[48](#_ENREF_48)**]** | Uganda | Campaign style | General population | Ful/Eco/Emp | - | $127.61 | 2,119 | 116 | TRNG, SNST, EQP, SUPL, TNSP ,PER OVHD ,ADMN M&E ,TEST |
| Kahn, 2011[[82](#_ENREF_82)] | Kenya | Campaign style | General population | Ful/Eco/Emp | $13.78 | - | NS | NA | TRNG, SNST, SUPL, TNSP ,PER |
|  |  |  |  |  | $57.93 | - | NS | NA |  |
|  |  |  |  |  | $44.47 | - | NS | NA |  |
| Kahwa, 2008 [[93](#_ENREF_93)] | Tanzania | Health facility | General population | Inc/Eco/Emp | $15.97 | - | 53,926 | NA | BLDG, FURN EQP, SUPL, VEH ,PER ,TEST |
| Korte, (2020) [[42](#_ENREF_42)] | Uganda | Health facility ANC/PMTCT | ANC Male partners | Ful/Fin/Emp | $31.20 | $582.60 | 187 | 10 | TRNG, EQP, SUPL, PER |
|  |  | Self-testing, home-based |  |  | $30.30 | $462.30 | 519 | 34 |  |
| Labhardt, 2014[[50](#_ENREF_50)] | Lesotho | Home-based | General population | Inc/Fin/Emp | 14.14 | 393.33 | 1,083 | 39 | SUPL TNSP, PER, TEST |
|  |  | Mobile service |  |  | 12.87 | 206.60 | 1,207 | 75 |  |
| Labhardt, (2019) **[**[94](#_ENREF_94)**]** | Lesotho | Home-based weekdays | General population | Inc/Fin/Emp | $10.12 | $322.76 | NS | NS | TRNG, MTG, EQP, SUPL, TNSP, PER ,TEST |
|  |  | Home-based-weekends | General population | Inc/Fin/Emp | $19.27 | $1017.41 | NS | NS |  |
| Lasry, 2019[[59](#_ENREF_59)] | Botswana | Home-based | General population | Ful/Eco/Emp | $54.10 | $773.70 | 12,415 | 870 | SNT, EQP, SUPL, TNSP, PER, ADMN ,TEST |
|  |  | Mobile service |  |  | $34.70 | $583.85 | 12,820 | 766 |  |
| Liambila, 2008[[72](#_ENREF_72)] | Kenya | Health facility | General population | Inc/Fin/Emp | $46.12 | - | 27 | NA | TRNG, SNT, MTG, SUPL ,PER ,SUPV TEST |
| Maheswaran, 2016[[44](#_ENREF_44)] | Malawi | Self-testing | General population at health facility-1 | Inc/Eco/Emp | $8.73 | $78.06 | 6,759 | 756 | TRNG, EQP, PER, OVHD, M&E |
|  |  | Self-testing | General population at health facility-2 |  | $12.25 | $88.57 | 5,372 | 743 |  |
|  |  | Self-testing | General population at health facility-3 |  | $10.32 | $32.81 | 9,488 | 2,984 |  |
|  |  | Self-testing | General population at the health facility |  | $10.18 | $113.04 | 15,190 | 1,367 |  |
| Maheswaran, (2017)[[112](#_ENREF_112)] | Malawi | Health facility | General population | Ful/Fin/Emp | - | $508.74 | NA | NS | TRNG, EQP, PER, OVHD, M&E |
| Meehan, 2017[[52](#_ENREF_52)] | South Africa | Campaign-style | General population | Inc/Fin/Emp | $48.85 | $723.11 | 1,909 | 128 | BLDG, UTL, COMM, OVHD, M&E |
|  |  | Mobile service |  |  | $23.94 | $1006.61 | 3,057 | 74 |  |
| Mangenah, 2019 [[45](#_ENREF_45)] | Malawi | Self-test, home-based | General population | Ful/Eco/Emp | $9.82 | - | 152,671 | - | TRNG, SNST, BLDG, STOR, EQP, SUPL , TNSP ,VEH ,PER ,TEST ,WST ,REC |
|  | Zambia |  |  |  | $14.23 | - | 103,589 | - |  |
|  | Zimbabwe |  |  |  | $13.84 | - | 93,459 | - |  |
| Medley, (2019)[[123](#_ENREF_123)] | South Africa | Health facility | ANC Male partners | Inc/Fin/Emp | $55.19 | $356.22 | 966 | 150 | NS |
|  |  | Home-based |  |  | $111.38 | $1,041.58 | 280 | 30 |  |
|  |  | Self-testing |  |  | $49.17 | $1,435.94 | 401 | 23 |  |
| Menzies, 2009[[60](#_ENREF_60)] | Uganda | Stand-alone | General population | Inc/Fin/Emp | 20.52 | 107.15 | 8,391 | 1,616 | TRNG, BLDG, EQP, SUPL, UTL ,VEH ,PER ,TEST |
|  |  | Health facility | General population |  | 12.44 | 45.91 | 21,755 | 5,872 |  |
|  |  | Home-based | PLHIV Partners |  | 14.75 | 246.75 | 1,861 | 80 |  |
|  |  | Home-based | General population |  | 8.83 | 174.62 | 38,799 | 2,072 |  |
|  |  | Stand-alone | General population |  | 31.64 | - | 6,227 | 1,511 |  |
|  |  | Health facility | General population |  | 15.69 | - | 18,428 | 5,807 |  |
|  |  | Home-based | PLHIV Partners |  | 15.49 | - | 1,916 | 101 |  |
|  |  | Home-based | General population |  | 9.81 | - | 44,523 | 2,350 |  |
| Mostert ,(2020) [[113](#_ENREF_113)] | South Africa | Self-testing | General population | Inc/Eco/Emp | $4.25 | - | 123,727 | NA | SNST, EQP, SUPL, TNSP, VEH PER, M&E, TEST, WST |
| Muhumuza, 2012[[61](#_ENREF_61)] | Uganda | Health facility | General population | Inc/Fin/Emp | $4.49 | - | 34,119 | 3,753 | NS |
|  |  | Home-based |  |  | $10.68 | - | 31,770 | 953 |  |
| Mulogo, 2013[[62](#_ENREF_62)] | Uganda | Health facility | General population | Inc/Fin/Emp | $6.07 | $82.10 | 454 | 36 | TRNG, BLDG, FURN, SUPL, TNSP, PER |
|  |  | Home-based |  |  | $4.75 | $51.92 | 444 | 45 |  |
| Mwenge, 2017[[73](#_ENREF_73)] | Malawi | Health facility | General population | Inc/Eco/Emp | $6.50 | $105.11 | 3,404 | 304 | TRNG, BLDG, STOR, EQP, SUPL, VEH, PER, TEST, WST |
|  | Zambia |  |  |  | $4.24 | $73.66 | 2,789 | 251 |  |
|  | Zimbabwe |  |  |  | $8.87 | $180.55 | 1,542 | 93 |  |
| Negin, 2009[[63](#_ENREF_63)] | Kenya | Home-based | General population | Inc/Fin/Emp | 8.18 | 116.80 | 2,780 | 209 | TRNG, TNSP, TEST |
| Nichols, (2020)[[99](#_ENREF_99)] | Malawi | Health facility | General population | Inc/Fin/Emp | $2.94 | $121.64 | 248.00 | 6 | TRNG, SNST, EQP, PER, OVHD |
|  |  | Health facility |  |  | $5.77 | $187.88 | 261.00 | 8 |  |
|  |  | Self-testing |  |  | $6.01 | $227.63 | 1,063 | 28 |  |
| Nichols, (2019) [[124](#_ENREF_124)] | Zambia | Health facility | General population | Inc/Fin/Emp | $2.25 | $70.85 | 6,728 | 214 | TRNG, SNST, EQP, PER, OVHD, TEST |
|  |  | Self-testing |  |  | $5.41 | $516.30 | 3,059. | 32 |  |
|  |  | Self-testing |  |  | $5.68 | $542.87 | 2,294 | 24 |  |
| Obure, 2012[[75](#_ENREF_75)] | Kenya | Health facility OPD | General population | Inc/Eco/Emp | $8.13 | $66.84 | 5,486 | 780 | TRNG, BLDG, FURN EQP SUPL, COMM, PER, TEST |
|  |  | Health facility HTC |  |  | $11.77 | $157.03 | 9,005 | 1,527 |  |
|  | Eswatini | Health facility OPD | General population |  | $7.96 | $48.92 | 4,872 | 1,851 |  |
|  |  | Health facility HTC |  |  | $9.65 | $46.58 | 6,061 | 2,698 |  |
| Obure, 2015[[74](#_ENREF_74)] | Kenya | Health facility | General population | Ful/Eco/Emp | $16.08 | $53.58 | NS | NS | BLDG, SUPL, PER, OVHD ADMN, TEST |
|  | Eswatini |  |  |  | $31.13 | $121.42 | NS | NS |  |
| Ochoa-Moreno, (2020)[[100](#_ENREF_100)] | Zimbabwe | Health facility | Pregnant women or women breastfeeding | Ful/Eco/Emp | $80.00 | - | 305.00 |  | PER, TEST, ARV REC |
| Orlando, 2010[[13](#_ENREF_13)] | Malawi | Health facility | Pregnant women or women breastfeeding | Inc/Fin/Emp | $66.62 | - | 6,500 | 1,371 | BLDG, FURN, VEH, PER, TEST ARV |
| Parker, 2015[[53](#_ENREF_53)] | Eswatini | Home-based | General population | Inc/Fin/Emp | $9.02 | $281.22 | 170 | 75 | SNT, FURN, EQP, COMM, TNSP, PER, TEST |
|  |  | Mobile service |  |  | $19.68 | $445.20 | 228 | 60 |  |
| Perchal, 2006[[76](#_ENREF_76)] | Ethiopia | Health facility | Pregnant women or women breastfeeding | Inc/Fin/Emp | $49.69 | - | NS | NA | SUPL, PER, TEST, OTH |
|  |  |  |  |  | $13.36 | - | NS | NA |  |
| Perez, 2016[[54](#_ENREF_54)] | South Africa | Mobile service | General population | Inc/Fin/Emp | $9.88 | - | 22,152 | 699 | SNST, EQP, COMM, TNSP, PER, TEST |
|  |  | Health facility |  |  | $9.69 | - | 17,678 | 807 |  |
|  |  | Home-based |  |  | $6.78 | - | 48,330 | 896 |  |
| Rutstein, 2013[[78](#_ENREF_78)] | Malawi | Health facility | PLHIV Partners | Inc/Fin/Emp | $16.04 | - | 2,436 | NA | TNSP, PER, M&E, TEST |
|  |  |  |  |  | $7.60 | - | 2,537 | NA |  |
|  |  |  |  |  | $3.38 | - | 1,207 | NA |  |
|  |  |  |  |  | $30.40 | - | 1,267 | NA |  |
|  |  |  |  |  | $15.20 | - | 1,320 | NA |  |
|  |  |  |  |  | $6.76 | - | 627 | NA |  |
| Settumba (2015), [[102](#_ENREF_102)] | Uganda | Health facility | General population | Inc/Eco/Emp | $2.22 | - | 5296 | NA | TRNG, BLDG, EQP, SUPL, UTL TNSP, PER, ARV |
|  |  |  |  |  | $1.82 | - | 4983 | NA |  |
|  |  |  |  |  | $3.72 | - | 746 | NA |  |
|  |  |  |  |  | $2.18 | - | 7 | NA |  |
| Shade, 2013[[79](#_ENREF_79)] | Kenya | Health facility | General population | Inc/Fin/Emp | - | $19.31 | NA | 4,135 | TRNG, BLDG, SUPL, SUPV OTH |
|  |  |  |  |  | - | $9.69 | NA | 3,429 |  |
| Sharma, 2014[[55](#_ENREF_55)] | South Africa | Mobile service | General population | Inc/Eco/Emp | $4.43 | $6.74 | 890 | 381 | NS |
|  |  | Home-based |  |  | $6.69 | $9.87 | NS | NS |  |
| Sharma, 2016[[80](#_ENREF_80)] | Kenya | Home-based | PLHIV Partners | Inc/Eco/Emp | $33.99 | - | NS | NA | BLDG, EQP, SUPL, TNSP, PER, OVHD, M&E, STRT |
|  |  |  |  |  | $38.43 | - | NS | NA |  |
|  |  |  |  |  | $40.23 | - | NS | NA |  |
|  |  |  |  |  | $15.25 | - | NS | NA |  |
|  |  |  |  |  | $15.65 | - | NS | NA |  |
|  |  |  |  |  | $17.17 | - | NS | NA |  |
| Smith, 2015[[64](#_ENREF_64)] | South Africa | Home-based | General population | Inc/Fin/Emp | $7.08 | $19.01 | NA | NA | MTG, BLDG, EQP, SUPL, TNSP, PER, OVHD, M&E, STRT |
| Tabana, 2015[[65](#_ENREF_65)] | South Africa | Health facility | General population | Inc/Eco/Emp | $30.60 | - | 3,818 | NA | TRNG, BLDG, EQP, VEH, PER, TEST |
|  |  | Home-based |  |  | $23.35 | - | 8,177 | NA |  |
| Terris-Prestholt, 2006[[103](#_ENREF_103)] | Uganda | Campaign-style | General population | Ful/Eco/Emp | $39.18 | - | 1,526 | NS | BLDG, EQP, SUPL, VEH, PER, OVH, STR |
| Terris-Prestholt, 2008[[81](#_ENREF_81)] | Zambia | Health facility | General population | Ful/Eco/Emp | $31.01 | $95.76 | 1,381 | 455 | TRNG, SNST, BLDG, EQP SUPL, VEH, PER STRT, OTH |
|  |  |  |  |  | $32.83 | $46.51 | 239 | 166 |  |
| Toure,(2013)[[104](#_ENREF_104)] | Namibia | Health facility | Pregnant women or women breastfeeding | Ful/Eco/Emp | $21.07 | - | NS | NS | TRNG, MTG, SUPL, TNSP, PER, OVHD, SUPV, TEST ARV |
|  | Rwanda | Health facility |  |  | $9.51 | - | NS | NS |  |
| Tumwesigye, 2010[[66](#_ENREF_66)] | Uganda | Home-based | General population | Inc/Eco/Emp | $7.51 | $148.40 | 52,342 | NS | SUPL, TNSP, PER, TEST |
|  |  |  |  |  | $8.34S | - | 238,290 | NS |  |
| Vyas, (2020a),[[105](#_ENREF_105)] | Tanzania | Health facility |  | Inc/Eco/Emp | $5.73 | $524.94 | 25,593 | 279 | TRNG, BLDG, EQP, SUPL, PER, OVHD, TEST |
| Vyas (2020B), [[105](#_ENREF_105)] | Malawi | Health facility | General population | Inc/Eco/Emp | $3.43 | $140.13 | 18,509 | 453 | TRNG, BLDG, EQP, SUPL, PER, OVHD, SUPV, TEST |

^a^Key populations in this study include Men who have sex with men and female sex workers

^b^Key populations in this study include male truckers and female sex workers

^1^Ful =Full costing, Inc=Incremental cost, Fin=Financial cost, Eco= Economic cost, Emp= Empirical (primary) cost, Mod=Modelled cost

^2^Training, workshops= TRNG, Sensitization, Events, Opening Ceremony, Outreach= SNST, Meeting=MTG, Building, Space, Building operation and maintenance, office rental= BLDG, Storage=STOR, Furniture= FURN Equipment, assets= EQP, Supplies= SUPL, Utilities= UTL, Communication, airtime, cell phones= COMM, Transport, travel= TNSP, Vehicle, bicycle, VEH operation and maintenance=VEH, Personnel, salaries, staff, labour, food/per diem=PER, Overhead, Central support costs=OVHD, Administration=ADMN, Monitoring & Evaluation, follow up, census, tracing=M&E, Supervision, auditing=SUPV, Test kits, viral load tests, testing commodities, diagnostics=TEST, Drugs, ARV, treatment commodities, medications=ARV, Waste Management=WST, Other start-up=STRT, Other recurrent=REC, Other (No specification)=OTH, Not applicable=NA, and Not specified=N
